# Fe(III)-(II) Mineral Transformation and Associated Organic Matter Stabilization Processes in a Tidal Marsh Soil

**DOI:** 10.1021/acs.est.6c05347

**Published:** 2026-08-03

**Authors:** Jan Jagode, Jannis F. Carstens, Hamed Kashi, Dörthe Holthusen, Kevin Tran, Heiner Fleige, Jana Carus, Elmar Fuchs, Franz Renz, Sandra Spielvogel, Georg Guggenberger

**Affiliations:** † Institute of Earth System Sciences, Section Soil Science, 26555 Leibniz University Hannover, Herrenhäuser Str. 2, Hannover 30419, Germany; ‡ 26534 Technische Universität Clausthal, Adolf-Roemer-Str. 2A, Clausthal-Zellerfeld 38673, Germany; § Institute for Plant Nutrition and Soil Science, 9179 Christian-Albrechts-University Kiel, Hermann-Rodewald-Str. 2, Kiel 24118, Germany; ∥ Department Ecological Interactions, 64346 Federal Institute of Hydrology, Am Mainzer Tor 1, Koblenz 56068, Germany; ⊥ Institute of Coordination Chemistry, 26555 Leibniz University Hannover, Callinstraße 3-9, Hannover 30419, Germany; # 26524 Technical University of Berlin, Institute of Ecology, Department of Plant Ecology, Rothenburgstraße 12, Berlin 12165, Germany

**Keywords:** anoxic soils, blue carbon, mineral-organic complexes, organic carbon stabilization, pyrite, siderite, stable carbon isotopes, tidal marsh

## Abstract

Iron (Fe) minerals are key agents in organic carbon (OC) stabilization in soils, yet their function under fluctuating redox conditions in tidal marshes remains poorly constrained, particularly for Fe­(II) phases. We conducted a six-month in situ incubation of synthetic mineral aggregates along a tidal marsh profile of the Elbe River, Germany, to examine Fe­(III)–Fe­(II) mineral interactions with OC under dynamic redox conditions. Membrane cylinders containing synthetic ferrihydrite, siderite, or pyrite aggregates with clay and sand were installed at four depths (15–60 cm). A subset received sorbed ^13^C-labeled reed (*Phragmites australis*) as dissolved and particulate organic matter to trace OC retention and redistribution. Sequential Fe extraction and Mössbauer spectroscopy showed progressive reduction of ferrihydrite and concurrent formation of siderite in deeper, anoxic layers. Organic matter enhanced Fe­(III) reduction and modulated mineral transformation in reducing zones to Fe­(II) minerals. In oxic horizons, ferrihydrite stabilized added reed-derived OC through classical surface sorption, while siderite and pyrite retained comparable or higher fractions of mineral-associated OC in deeper, anoxic horizons, primarily within the aggregate, with partial redistribution to surrounding soil. These results demonstrate that Fe­(II) minerals non-negligibly contribute to OC stabilization under reducing conditions, a mechanism likely to intensify with ongoing sea-level rise.

## Graphical abstract

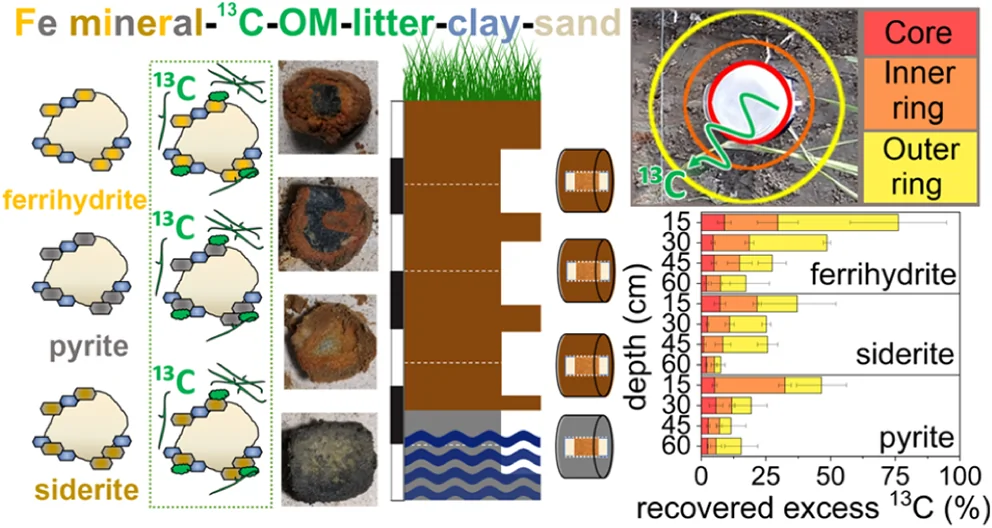

## 1 Introduction

Sorption of dissolved organic matter (DOM) onto Fe (oxyhydr)­oxides—hereafter referred to as Fe­(III) oxides—plays a critical role in stabilizing organic carbon (OC) in soils.^1,2^ However, in tidal marsh soils, periodic inundation and redox fluctuations strongly influence mineral-OC interactions and, consequently, OC stabilization.^3,4^ Under reducing conditions, Fe­(III) oxides are reductively dissolved through microbial organic matter (OM) degradation,^5,6^ leading to the disintegration of mineral aggregates and the release of previously sorbed OM into the soil solution, thereby promoting OC mobilization.^7,8^ Under anoxic and reducing conditions, common in coastal and estuarine environments, the effectiveness of Fe­(III) oxides as OC binding agents is thus likely limited and yet still not well understood.^9,10^ In these environments, high bicarbonate and sulfide concentrations, along with riverine phosphate inputs and reductive dissolution of Fe­(III) oxides, favor the formation of secondary Fe­(II) minerals such as siderite (FeCO_3_), iron sulfides, e.g., pyrite (FeS_2_) or vivianite (Fe_3_(PO_4_)_2_·8H_2_O).^3,11,12^ Thus, a notable fraction of Fe persists as solid Fe­(II) phases. The formation of Fe­(II) minerals will likely increase in the future as a consequence of sea-level rise and salinity increases.^3,13^

To date, most in situ work has focused on Fe oxide transformation and stability in freshwater and semiterrestrial systems—including rice paddies, river floodplains, and forested hydromorphic soils,^7,9,14–18^ with few studies in coastal settings.^11,19^ Consequently, our understanding of Fe­(III) oxides and their associated OC persistence under fluctuating redox conditions in coastal soils, along with the potential of Fe­(II) minerals to stabilize OC, is limited.^3,20,21^ While tidal marshes contain high biomass productivity and topsoil OC stocks, these are not necessarily indicative of long-term carbon sequestration.^22^ Long-term stabilized OC is typically found at greater depths—under more reducing conditions—and originates predominantly from allochthonous sources.^22–24^

To address the role of different Fe minerals in stabilizing OC under fluctuating redox conditions, we investigated how Fe­(II) and Fe­(III) minerals transform and persist in the fluctuating water-saturated high marsh soil of the tidal Elbe estuary and how these changes are related to OC bound to the different Fe minerals, as well as its redistribution and mobility along redox gradients. In a novel approach, we combined synthetic Fe­(III) and Fe­(II) aggregates and ^13^C isotopic tracing in a field incubation experiment. Membrane cylinders containing artificial Fe mineral-clay-sand aggregates (Fe-MCS) and Fe mineral-organic-clay-sand aggregates (Fe-MOCS) with the Fe minerals ferrihydrite (Fh), pyrite (Py), and siderite (Sd) were buried at four depths within a tidal marsh soil profile. In Fe-MOCS, ^13^C-enriched OM was sorbed to Fe-MCS and supplemented with ground reed (*Phragmites australis*) litter as a particulate OM source and electron donor. This setup enabled the assessment of OC retention and exchange with the surrounding soil. We applied sequential Fe extraction and Mössbauer spectroscopy to investigate mineral phase transformations and stable isotope analysis to trace ^13^C-OC stabilization by the different Fe minerals and along their transformation in oxic and anoxic soil layers.

We hypothesized that (i) under field conditions shaped by fluctuating tidal inundation, groundwater dynamics, redox potential, and salinity, both Fe­(II) and Fe­(III) minerals function as inorganic binding agents and OC sinks; (ii) Fe­(III) oxides dissolve in the reduced lower part of the profile and promote hotspots for Fe­(II) mineral formation; and (iii) siderite- and pyrite-coated aggregates remain stable under reducing conditions, along with their associated OC, but transform into Fe­(III) oxides in oxic surface layers.

## 2 Materials and Methods

### 2.1 Study Site and Profile Description

The field incubation experiment was conducted in the high marsh, defined here as the elevated part of the tidal marsh that is only episodically inundated during storm tide events and characterized by mesophilic grassland vegetation. The study site was located near Krautsand along the tidal Elbe River (53.7648, 9.3756; Elbe-km 674.2), downstream of Hamburg, Germany (Figure 1). The site was selected during the 2020 Uferfunk campaign,^25^ with initial soil analysis (Table 1, Text S1). The soil profile was classified as a Calcaric Fluvic Tidalic Gleysol according to the World Reference Base for Soil Resources.^26^ The AhBl horizon is predominantly oxic, but episodically reduced during flooding. The underlying Bl1 and Bl2 horizons experience fluctuating redox conditions (oxic to anoxic) driven by groundwater table dynamics and tidal inundation, as reflected by redoximorphic features. The Brl horizon remains predominantly reduced under long-term anoxic conditions. The Krautsand high marsh zone is episodically flooded during storm floods (∼17 events yr^–1^) based on elevation and the DGM-W model.^27^ The field incubation was conducted from August 2021 to March 2022, spanning a dry summer to wet winter transition.

**Table 1:** Physicochemical Properties of Soil Horizons at the Tidal Marsh Study Site (Sampled in 2020), Including Electrical Conductivity (EC) and Organic Carbon (OC)^a^

**horizon**	**depth (cm)**	**sand (%)**	**silt (%)**	**clay (%)**	**EC**(μs cm^–1^)	**pH**	**OC (%)**	**δ^13^C_org_ (‰)**	**CaCO_3_ (%)**	**Fe_d_** (g kg^–1^)	**Fe_o_** (g kg^–1^)
AhBl	0–20	11.6	69.2	19.2	611	7.4	3.00	–27.94	3.55	13.6	10.3
Bl1	20–35	45.0	46.0	9.0	280	7.6	1.55	–26.87	4.39	7.6	6.3
Bl2	35–65	42.5	44.0	13.5	269	7.7	1.38	–26.45	3.54	8.4	6.3
Brl	65–100	96.9	1.9	1.3	212	7.2	0.07	–26.76	0.78	1.6	3.2

a Horizons were defined according to the IUSS Working Group WRB (2022).

**Figure 1:**
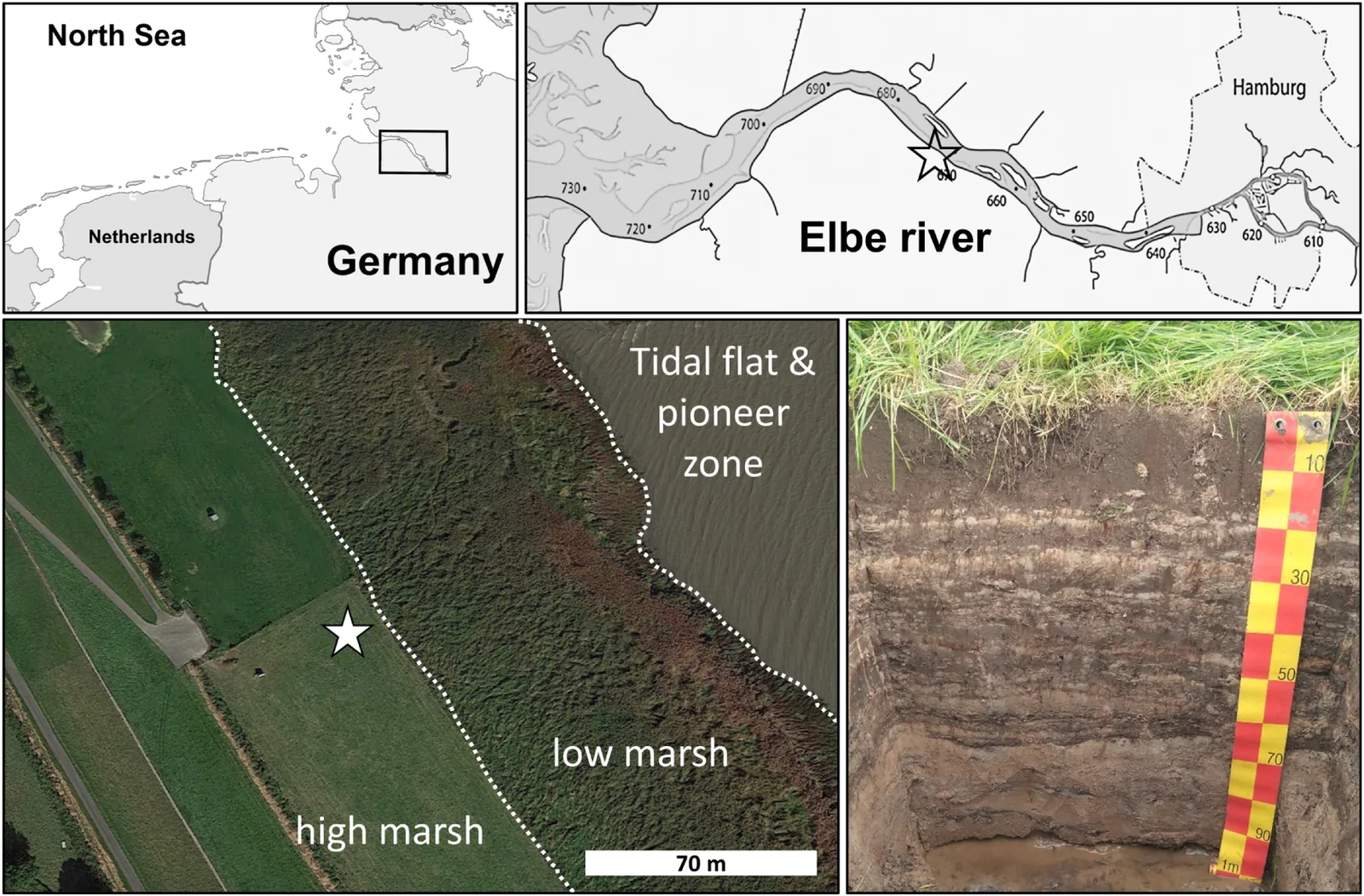
Location and site characteristics of the field experiment in the Elbe River tidal marsh. The star marks the location of the field incubation in the high marsh site near Krautsand (Lower Saxony, Germany). Aerial photograph source: Landsat/Copernicus.

### 2.2 Field Incubation Experiment

A field incubation experiment was designed to investigate how different Fe mineral types (Fe­(III) oxides and Fe­(II) minerals) affect OC stabilization under natural redox gradients in this tidal marsh soil (Figure 2). By comparing unamended (Fh-MCS, Sd-MCS, and Py-MCS) and ^13^C-OM-amended (Fh-MOCS, Sd-MOCS, and Py-MOCS) aggregates in membrane cylinders across depths, mineral-specific Fe transformation and OC redistribution and retention were assessed. Minerals were synthesized and verified by XRD without sand and clay additions (Text S2, Figure S1).

**Figure 2:**
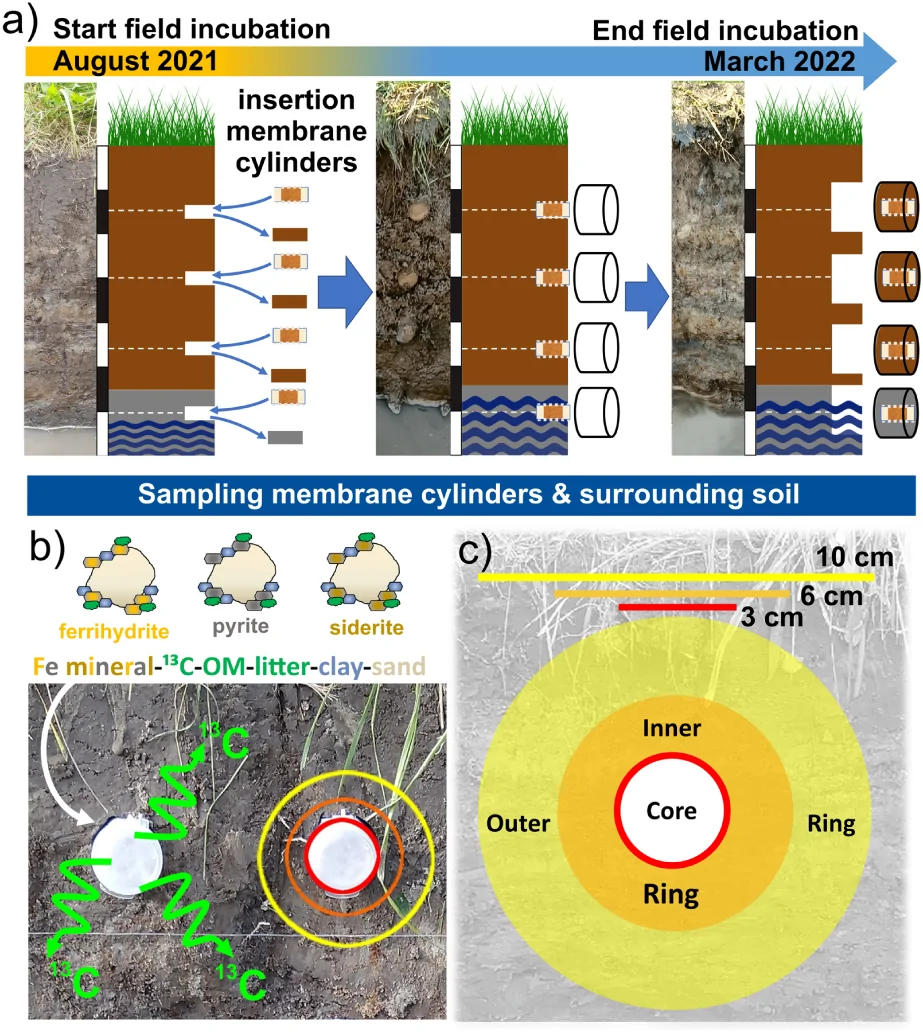
Field setup and sampling design for Fe mineral–^13^C organic matter (OM)–litter–clay–sand aggregate incubation (a). Membrane cylinders containing aggregates (ferrihydrite, pyrite, or siderite with sorbed ^13^C-labeled reed organic matter and reed litter) were inserted at multiple soil depths in August 2021 and retrieved in March 2022. Surrounding soil was sampled in concentric zones (core: 0–3 cm, inner ring: 3–6 cm, outer ring: 6–10 cm) to assess spatial ^13^C distribution and carbon stabilization (c).

#### 2.2.1 Ferrihydrite–Illite–Sand Aggregates

Ferrihydrite synthesis was adapted from Schwertmann and Cornell.^28^ 500 g of quartz sand (G0-03T, Schlingmeier, Germany) and 20 g of illite (Mica SFG70, Aspanger, Austria, <2 μm) were mixed and shaken with a vortex (Text S3). Added to this, 500 mL of 0.5 M FeCl_3_ was adjusted via dropwise addition of 10 M NaOH to pH 7 while stirring vigorously. The aggregates were filled into dialysis tubing (MWCO 1000) and dialyzed against bidest. water until the electrical conductivity of the supernatant was <10 μS cm^–1^. The ferrihydrite–illite–sand aggregates were shock-frozen via dropwise addition to liquid N_2_ and freeze-dried for 7 days. The dried product was sieved to >63 μm. The final target clay content in the aggregates was ∼1%, similar to that in the lowermost horizon. The product was stored in sealed glass bottles at room temperature (RT) until used.

#### 2.2.2 Pyrite–Illite–Sand Aggregates

Pyrite synthesis was carried out by dissolving FeCl_3_·6H_2_O in 500 mL of deaerated, acetic acid buffer within a glove bag to obtain a 0.5 M FeCl_3_ solution.^29^ To this, 500 g of quartz sand and 20 g of illite were added and subsequently stirred. Then, 500 mL of 1.5 M polysulfide solution was slowly added while vigorously stirring (both manually and magnetically). The slurry was stirred at RT for 1 h and then at 90–100 °C for 4 h. Thereafter, the slurry was filled into reaction bottles, sealed airtight, and aged in an oven at 80 °C for 7 days. The precipitate obtained was centrifuged and washed with deaerated bidest. water, 10% HCl (to remove any remaining FeS), and bidest. water again until the supernatant was clear and pH neutral (checked with pH indicator strips). The pyrite–illite–sand aggregates were shock-frozen via dropwise addition to liquid N_2_ and freeze-dried for 7 days. The dried product was sieved to >63 μm. The final clay content was ∼1%. The product was stored under N_2_ atmosphere and used within two months.

#### 2.2.3 Siderite–Illite–Sand Mixture

Siderite was synthesized by mixing deaerated equimolar solutions (2 M) of ferrous chloride and sodium carbonate in a Captair pyramid glovebag under N_2_.^30^ The reaction bottle was sealed to exclude oxygen and then stirred for 24 h outside the glovebag at RT. After aging for 3 days, the precipitate was centrifuged and washed with deaerated bidest. water several times to get rid of excess salt. Siderite was shock-frozen via dropwise addition to liquid N_2_ and freeze-dried for 7 days. Contact with air was minimized as much as possible. Twenty grams of siderite were added to a mixture of 500 g of quartz sand and 4 g of illite (∼1%) and then homogenized inside the glovebag. The product was stored under N_2_ atmosphere and used within two months.

#### 2.2.4 Organic Matter Coating of Minerals with ^13^C-labeled Dissolved Organic Matter

For the organic matter coating on the Fe minerals, ^13^C-labeled DOM was prepared by extraction of a mixture of 100 g labeled dried, ground reed (*Phragmites australis*) plants (δ^13^C 258.39 ‰) and 100 g of dried, ground natural reed (δ^13^C −27.0 ‰) in 1000 mL bidest. water to limit the need for expensive labeled plant matter. The mixture was shaken for 16 h at room temperature. Coarse reed material was removed via sieving, and the remaining solution was prefiltered by pressure filtration through glass fiber filters (GF 92, Whatman, Chicago, USA) and finally pressure filtered through 0.45-μm cellulose nitrate filters (Sartorius, Göttingen, Germany). The bulk solution had a concentration of 4744 ± 23.0 mg C l^–1^ and δ^13^C 471.8 ‰. A 560 g of Fe mineral–illite–sand aggregates were submerged in 180 mL ^13^C-labeled reed DOM solution and left to stand for 60 min, then filtered, and rinsed with bidest. water to remove nonsorbed OM. Subsequently, 12 g of finely ground, unlabeled reed (see S4) was added to mimic plant residue input and to provide electron donors. The mixture was stirred until homogenized (equivalent to the final OC content of ∼1%, like the soil background).

#### 2.2.5 Field Incubation Setup and Analysis

Membrane cylinders (PA-15/10, Franz Eckert GmbH, Waldkirch, Germany) were filled with three layers (inert quartz sand–45 g Fe-MCS or 45 g Fe-MOCS–inert quartz sand), ensuring that only Fe mineral aggregates (Table 2) were in contact with the membrane. During storage and transport, membrane cylinders were kept in airtight containers, refrigerated, and maintained under inert gas (see Text S4 for details). At the Krautsand field incubation site, a soil profile was dug, the profile wall was perforated with a pipe, and membrane incubation cylinders were laterally inserted into the holes at four depths (15–60 cm, in 15 cm increments) in three replicates, with the deepest positioned just below the groundwater level. The profile was closed again, excavated soil was backfilled according to the original horizon sequence, and membrane incubation cylinders were incubated in situ for 6 months. Afterward, the soil profile wall was carefully excavated until the tops of all membrane cylinders were exposed. Subsequently, 10 cm diameter PP containers were centered over each membrane cylinder and pressed into the soil profile wall, and membrane cylinders were recovered together with the surrounding field-moist soil, sealed with a lid, and enclosed in PE bags for transport. Sampling containers were stored at ambient temperatures overnight and transported to the lab within 20 h, where they were subsequently stored in airtight containers at 4 °C until further processing. During processing, samples were divided into the outer ring (OR, 10–6 cm diameter), inner ring (IR, 6–3 cm diameter), and core (membrane cylinder filling, 3 cm diameter). Membrane cylinder fillings were shock-frozen in liquid N_2_, cut into aliquots with a steel saw (sample surfaces were cleaned to avoid contamination), and stored under N_2_ atmosphere until analysis.

**Table 2:** Fe Content, Organic Carbon (OC) Concentration, and δ^13^C Values in Fe Mineral-Organic-Clay-Sand of Ferrihydrite, Siderite, and Pyrite (Fh-MOCS, Sd-MOCS, Py-MOCS) Prior to Field Incubation^a^

Aggregate	Fe (mg g^–1^)	OC (%)	δ^13^C (‰)
Fh-MOCS	12.17	1.2	–6.21
Py-MOCS	5.1	1.32	7.59
Sd-MOCS	11.34	1.25	–1.04

a Due to the HCl washing step, Py-MOCS Fe contents were lower than those of Fh-MOCS and Sd-MOCS.

Organic C content and δ^13^C were determined analogous to the basic soil samples (Text S1). Atom% ^13^C was calculated as follows:

1 $$\mathrm{atom\%}=\frac{100\times 0.0111802\times \left(\frac{\delta^{13}C}{1000}+1\right)}{1+0.0111802\times \left(\frac{\delta^{13}C}{1000}+1\right)}$$

with 0.0111802 as the standard isotope ratio of Vienna Pee Dee Belemnite; VPDB. For calculation of excess ^13^C values of Fe-MOCS in membrane incubation cylinders (Table S1), we used the following equation:

2 $${\mathrm{excess}}^{13}C=\frac{{\mathrm{atom\%}}_{\mathrm{labeled}}-{\mathrm{atom\%}}_{\mathrm{unlabeled}}}{100}\times {\mathrm{OC}}_{\mathrm{labeled}}$$

To compare excess ^13^C recovery between Fe-MOCS in membrane cylinder core, the inner ring (IR), and the outer ring (OR) of the surrounding soil, differences in sampling volume had to be accounted for.

C13recovery(%)=excess13Cfractionexcess13Cpreinc×VfractionVpreinc×100 3

The core, IR, and OR zones had volumes of V_preinc_ = V_core_ = 70.7, V_IR_ = 212.1, and V_OR_ = 502.7 cm^3^, where V_preinc_ refers to the preincubation core volume that was initially ^13^C-labeled. Consequently, larger zones could dilute the measured ^13^C signals. To correct for this, the excess ^13^C concentrations in the IR and OR were volume-normalized using correction factors of 3 (IR) and 7.1 (OR), relative to the core. Soil bulk density was assumed to be uniform across all zones, allowing for a direct comparison of ^13^C recovery across the profile.

### 2.3 Sequential Fe Extraction

The different Fe mineral–illite-coated sand samples were analyzed for different Fe species by sequential Fe extraction in field-moist samples based on Lenstra et al.^31,32^ The sequential extraction steps are (1) ascorbic acid-citrate-bicarbonate (pH 7.5) to extract poorly ordered Fe oxides such as ferrihydrite (Fe_Asc_); (2) 1 M HCl to dissolve carbonates and FeS (Fe_HCl_); (3) citrate-buffered dithionite to extract crystalline metal oxides (Fe_Dith_); (4) acid ammonium oxalate to extract magnetite (Fe_Oxa_); and (5) concentrated HNO_3_ to extract pyrite $\left(Fe_{HNO_{3}}\right)$ . For dithionite extraction, the 16 h method^33^ was employed instead of the heated Mehra–Jackson method.^30,34^ Both methods have been shown to be in close agreement.^35^

For 2 g of field wet soil, the first 4 sequential steps employed 40 mL of extraction solution and the HNO_3_ step used 10 mL, which was afterward diluted to 40 mL. All solutions were flushed with N_2_ before being added to the samples, except for the concentrated HNO_3_ solution. During the extraction, the samples were shaken on an overhead shaker in the dark. After extraction, solutions from steps 1–4 were centrifuged (15 min, 4000 g). The concentrated HNO_3_ solution was passed through a 0.45 μm PTFE filter (Fisher Scientific GmbH, Schwerte, Germany). Concentrations of Fe were analyzed by a modified ferrozine assay.^36^

### 2.4 Mössbauer Spectroscopy

Mössbauer ^57^Fe spectra were recorded in transmission mode with a modified Mössbauer spectrometer, produced by Wissenschaftliche Elektronik GmbH (Starnberg, Germany)^37^ utilizing a ^57^Co/Rh source at RT. Velocity calibration was based on an α-Fe reference. Aggregate samples were measured with a layer thickness of 1–2 mm. The data were fitted by a least-squares method using Lorentzian-shaped lines with Recoil Mössbauer Analysis software.^38^

### 2.5 Data Analysis

Two-way ANOVA tested for effects of Fe mineral and depth on OC and excess ^13^C; three-way ANOVA additionally included sampling zone. Tukey–Kramer tests (*p* < 0.05) were used for pairwise comparisons. Analyses were performed in OriginLab Pro 2023 (OriginLab, Northampton, Massachusetts, USA).

## 3 Results and Discussion

### 3.1 Fe Mineral Extractabilities by Different Extractants

Visual inspection of the incubated Fe mineral–clay–sand aggregates revealed distinct color changes after the in situ incubation, indicative of redox-driven transformations (Figure 3). In particular, siderite-coated sands (Sd-MCS) developed reddish hues in upper layers, suggesting oxidation. Fe-mineral aggregates amended with organic carbon (Fe-MOCS) showed more pronounced transformations and spatial redistribution, as evidenced by the variety of color shifts across depths. Fh-MOCS showed increasing brown-to-black color changes—particularly in areas without direct soil contact, which turned green-black in the lowermost horizon. Py-MOCS showed persisting black colorations aside from the uppermost horizon shifting to gray and surficial reddish-brown colorization in areas with soil contact. Sd-MOCS shifted toward reddish-brown colors with either black or discolored areas; within Sd-MOCS, the lowermost horizon changed to green-black.

**Figure 3:**
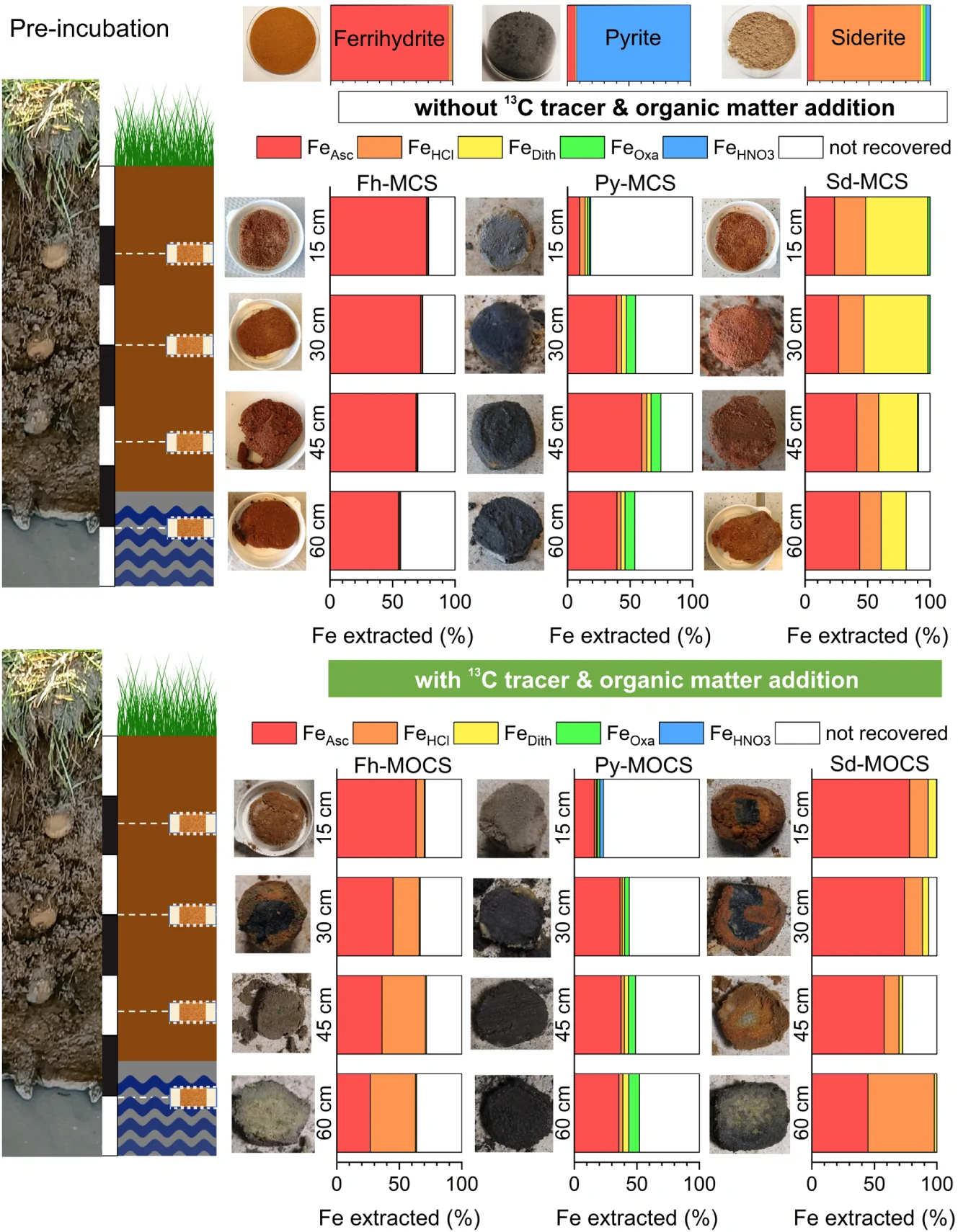
Sequential Fe extraction of Fe mineral–illite–sand aggregates compared to preincubation Fe content.

In the unamended Fh-MCS, nearly all reactive Fe was recovered as Fe_Asc_ (>96%) across all depths (Figure 4), indicating that ferrihydrite remained the dominant phase. Total Fe recovery (over all extraction steps, Fe_seq_) relative to that at preincubation was high in upper soil layers (79.0 ± 6.1% at 15 cm) but decreased with depth to 56.5 ± 11.9% at 60 cm (Figure 3), suggesting progressive dissolution or redistribution with soil depth. The addition of OM in Fh-MOCS showed a clear shift from Fe_Asc_ to Fe_HCl_, which had already started at 30 cm soil depth and increased with further depth, indicating reductive transformation into Fe­(II) phases (Figure 4). Fe recovery as the original Fe phase (Fe_Asc_) decreased from 63.5 ± 6.0% at 15 cm to 26.8 ± 2.7% at 60 cm (Figure 5).

**Figure 4:**
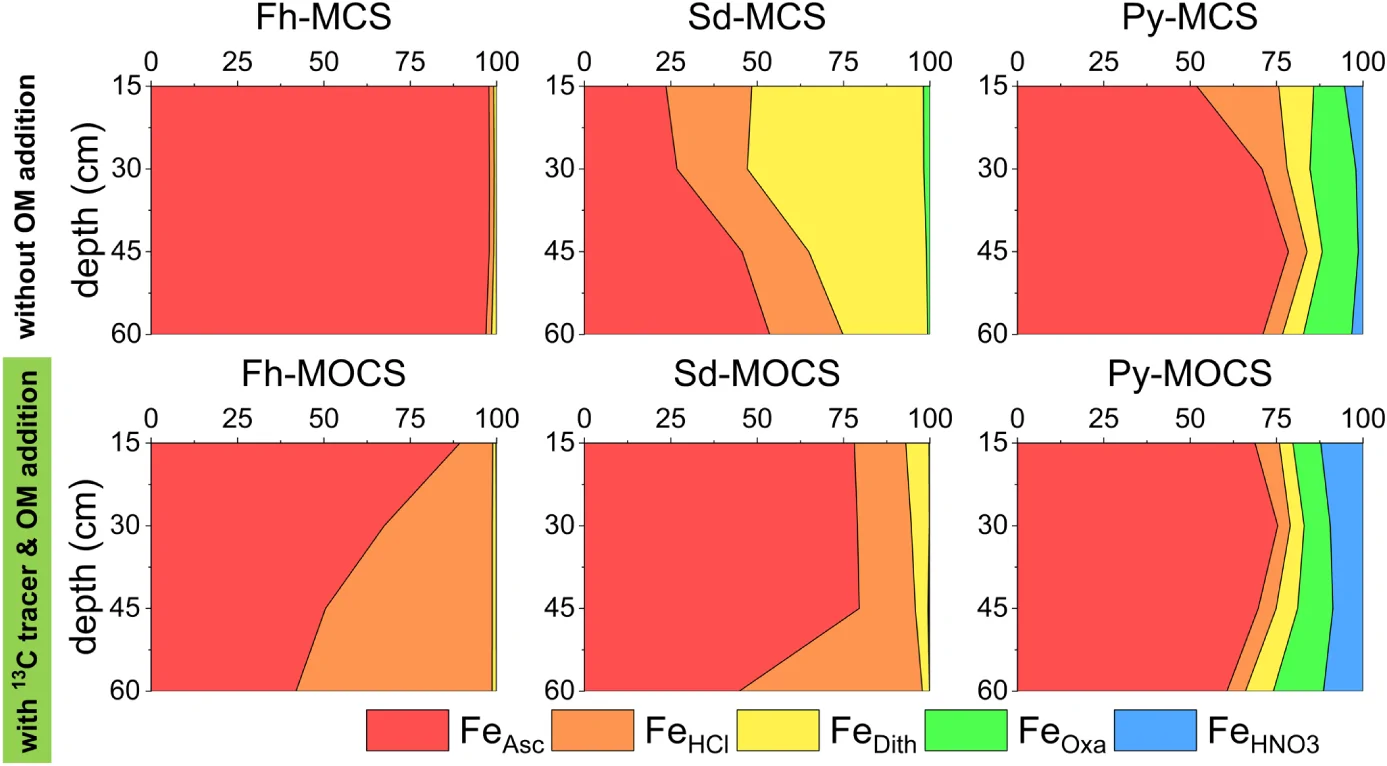
Postincubation composition of reactive Fe fractions according to sequential Fe extraction in ferrihydrite (Fh), pyrite (Py), and siderite (Sd) mineral aggregates without (Fh-MCS, Py-MCS, Sd-MCS) and with organic matter addition (Fh-MOCS, Py-MOCS, Sd-MOCS).

**Figure 5:**
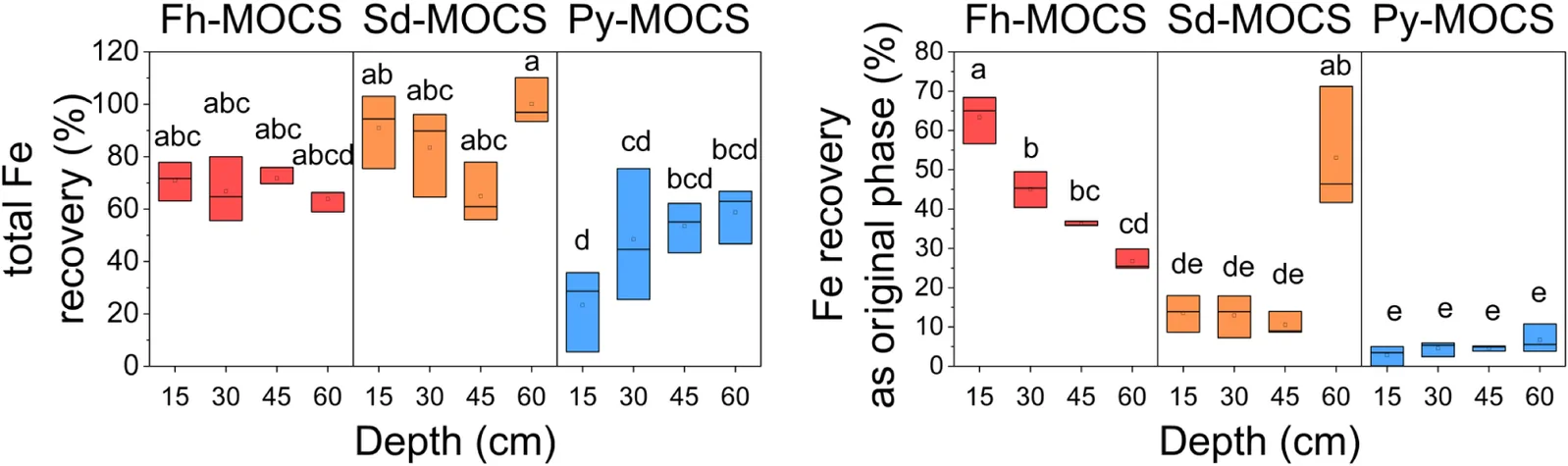
Fe recovery from Fe-mineral–organic clay–sand aggregates (MOCS) after 6-month incubation in tidal marsh soil based on sequential extraction. Left: total Fe recovery relative to initial Fe content across depths. Right: Fe recovery as the original mineral phase (ferrihydrite, siderite, pyrite).

The pyrite-based aggregates (Py-MCS and Py-MOCS) retained only minor fractions of $Fe_{HNO_{3}}$ in the uppermost soil depth (<7%), the main fractions being primarily Fe_Asc_ and secondarily Fe_Oxa_, with minor contributions from Fe_HCl_ and Fe_Dith_. Concurrently, both Py-MCS and Py-MOCS variants showed higher Fe losses than other mineral types, with total Fe recovery ranging from 23.3 to 58.8% for Py-MOCS and 18.6–75.7% for Py-MCS. Together, this indicates instability of this Fe­(II) phase, particularly under the oxic conditions in the topsoil, and redistribution of Fe phases.

In the OM-free siderite aggregates (Sd-MCS), Fe was mainly recovered as Fe_Asc_, Fe_HCl_, and Fe_Dith_. With increasing soil depth, Fe_Dith_ decreased, while Fe_Asc_ increased. Fe_HCl_ remained relatively constant across depths at 17.3–27.4%. In Sd-MOCS, Fe_Dith_ represented only a minor portion (<7%). Here, Fe_Asc_ dominated the upper horizons (79.7 ± 12.2% at 15 cm). However, with increasing soil depth, Fe_Asc_ declined, while Fe_HCl_ increased, comprising the majority of reactive Fe at 60 cm (59.6 ± 17.8%). Recovery of Fe content was notably high, with ranges of 72.8–112.4% for Sd-MOCS and 81.5–110.3% for Sd-MCS (Figure 5).

To assess Fe species distribution in Elbe tidal marsh soils, a five-step sequential Fe extraction was applied.^31,32^ Common Fe minerals observed in tidal wetlands are ferrihydrite, lepidocrocite, goethite, pyrite, siderite, and vivianite, while jarosite and schwertmannite can occur due to drainage and oxidation of Fe­(II) sulfides, leading to acid sulfate soil development.^3^ Reference extractions showed high step-specific efficiencies (generally >87%) for ferrihydrite, goethite, siderite, pyrite, vivianite, and others. Some lower target recoveries (53–71%) were observed for phases such as mackinawite, lepidocrocite, low-crystalline schwertmannite, and fine magnetite, which were partially reactive across multiple reagents (Table S3). In Elbe tidal marsh soil samples, extractable Fe typically followed the pattern: Fe_Asc_ ≥ Fe_HCl_ > Fe_Dith_ > $Fe_{HNO_{3}}$ > Fe_Oxa_. Thus, Fe fractions indicate a dominant presence of short-range ordered Fe­(III) oxides and Fe­(II) carbonate/sulfide phases, with smaller contributions from crystalline Fe­(III) oxides, pyrite, and mixed-valence phases.^31^

A critical aspect of sequential extraction is that each reagent step removes its targeted Fe phase, preventing its later coextraction in subsequent steps. This enables clearer phase differentiation than single-step Fe_o_ and Fe_d_ extractions commonly used in terrestrial soil studies. Although these single-step methods are widely applied to quantify reactive Fe pools, they often distort oxidation-state information and produce overlapping responses. The necessity for sequential extraction originates from the partial extraction of Fe­(II) minerals, leading to phase misinterpretation. Oxalate dissolves siderite almost completely, whereas dithionite removes only 18–22% in single-step extractions.^39,40^ Mackinawite is approximately 48% extractable by dithionite. Vivianite is more than 90% dissolved by oxalate, but less by dithionite. Magnetite is nearly completely dissolved by oxalate (73–100%) but only marginally by dithionite (∼5%).^40^

Data on Fe­(II) minerals in common single-step extractions remain sparse.^41^ In contrast, sequential extractions fractionate the Fe pool, offering more specificity but also requiring stepwise interpretation and possible cross-contamination. FeS was partially extracted, and vivianite was fully extracted in the Fe_Asc_ step, even though FeS was intended to be targeted by the subsequent Fe_HCl_ extraction.^31^

Further, Fe-OM associations may influence the outcome of Fe extraction methods due to both chemical and structural effects.^14^ When Fe is coprecipitated or adsorbed with OM, the resulting complexes often exhibit higher resistance to reductive dissolution, particularly in dithionite extractions.^42^ This is because OM can form protective coatings or inner-sphere complexes that shield Fe oxides from reductants, thereby reducing extractability. For example, ferrihydrite coprecipitated with organic ligands, such as low-molecular-weight organic acids, has been shown to dissolve more slowly under reductive conditions compared to pure mineral phases.^10^ OM-Fe associations can affect the crystallinity and reactivity of the Fe phases themselves, often resulting in poorly ordered or amorphous structures. Protective layers may respond differently to standard extractants and further affect the transformation pathways and stability. OM coatings likely influenced the observed Fe fractionation by reducing extractant accessibility through surface shielding and inner-sphere complexation, particularly for short-range ordered Fe­(III) oxides targeted by Fe_Asc_.^42,43^ In addition, Fe-OM coprecipitation can produce structurally modified, poorly crystalline phases with altered dissolution kinetics.^10,14^ With the onset of suboxic to anoxic conditions at lower depths and increasing water saturation, Fe­(III) oxides were progressively reduced and dissolved, diminishing their capacity to stabilize OM and promoting OC mobilization. Simultaneously, these reductive processes created local hot spots for secondary Fe­(II) mineral formation, particularly siderite, indicating an increasingly important role for Fe­(II) minerals in deeper soil zones.

### 3.2 Fe Mineral Transformation in Tidal Marsh Soils

Mössbauer spectra revealed three to four consistent Fe mineral phases in both Fh-MOCS and Sd-MOCS (Table S3). In contrast, for Py-MOCS, a reliable spectral deconvolution was not possible due to significant signal overlap between Fe sulfide phases and Fe­(III) in clay, an issue previously reported.^44^ The isomer shift (δ) and quadrupole splitting (ΔE_Q_) of the recurring observed phases correspond to δ = 0.39 ± 0.04 and ΔE_Q_ = 0.84 ± 0.12, δ = 1.20 ± 0.02 and ΔE_Q_ = 1.90 ± 0.08, δ = 1.20 ± 0.05 and ΔE_Q_ = 2.45 ± 0.15, and δ = 0.25 ± 0.04 and ΔE_Q_ = 0.39 ± 0.07 (Figure 6). These match well with data given for the expected occurrence of ferrihydrite, siderite, Fe­(II) in illite, and Fe­(III) in illite according to the literature.^45–47^ While Mössbauer site population fractions are only semiquantitative, owing to differing recoil-free fractions of Fe­(II) and Fe­(III), they nevertheless reveal clear qualitative trends that confirm previous observations of the sequential extraction. In both Fh-MOCS and Sd-MOCS samples, the relative proportion of Fe­(III) in ferrihydrite decreased with soil depth, whereas the signal assigned to Fe­(II) in siderite increased progressively (Figure 7).

**Figure 6:**
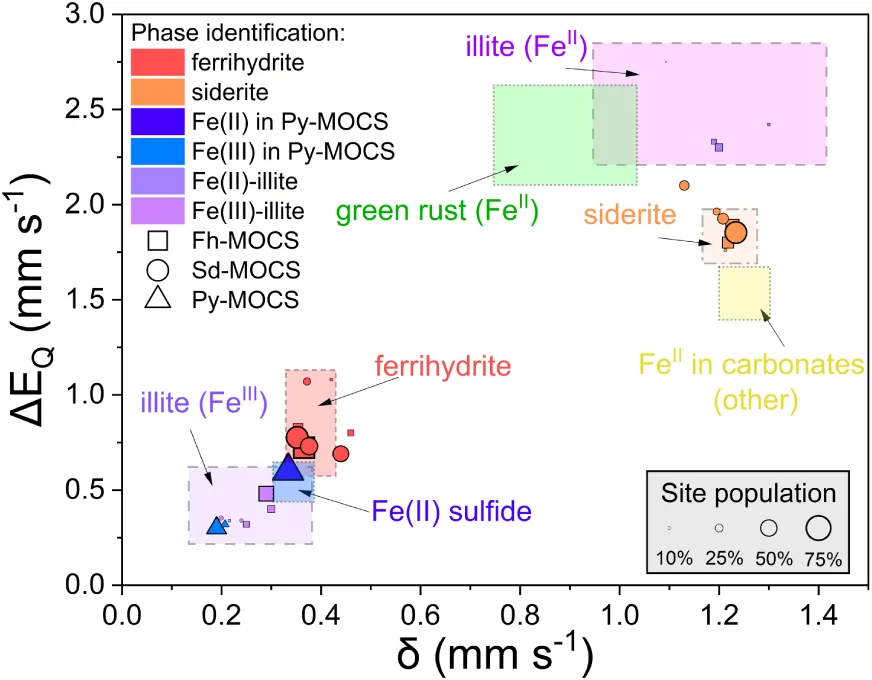
Mössbauer isomer shift (δ) and quadrupole splitting (ΔE_q_) of ferrihydrite-based mineral–organic clay–sand aggregates (Fh-MOCS), siderite-based MOCS (Sd-MOCS), and pyrite-based MOCS (Py-MOCS) at 15–60 cm depth after 6-month incubation in tidal marsh soil. Colored fields show literature ranges of corresponding Fe minerals; symbol size indicates site population.

**Figure 7:**
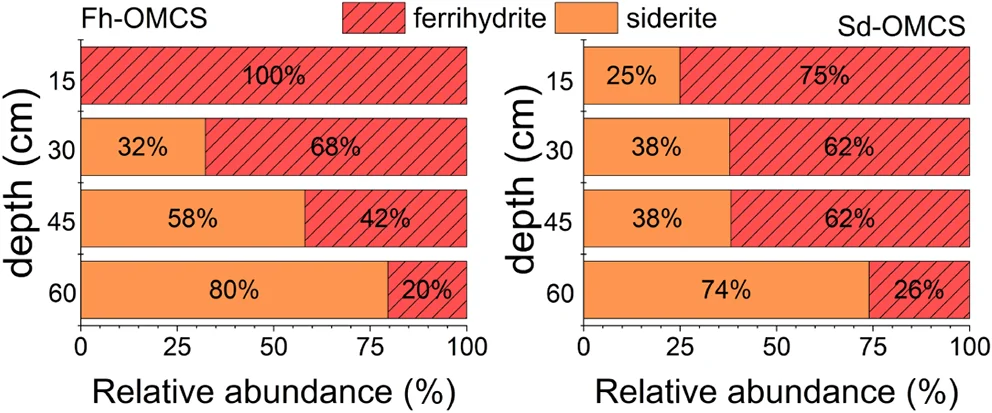
Depth-dependent changes in Mössbauer-resolved Fe site populations in ferrihydrite-based mineral–organic–clay–sand aggregates (Fh-MOCS) and siderite-based MOCS (Sd-MOCS). Site populations are normalized after excluding clay-associated Fe sites and are interpreted semiquantitatively. The figure summarizes the dominant depth-dependent trend from ferrihydrite-associated Fe­(III) toward siderite-associated Fe­(II).

The Fe mineral composition identified via the sequential Fe extraction (Figure 4) is largely corroborated by Mössbauer spectroscopy (Figures 6 and 7), which confirmed both the expected mineral phases and the observed ferrihydrite–siderite depth gradient, supporting mineral interpretation.

Fh-MCS without OM addition underwent minimal phase transformation within the whole soil profile, i.e., ferrihydrite remained the prevalent Fe phase. However, Fe loss occurred from cores, even in the upper soil layers. Fe loss likely resulted from (i) leaching of Fe-coated particles or (ii) dissolution and redox-driven mobilization of Fe into the soil solution.^9,48^ Under strong redox fluctuations, dissolution–reprecipitation cycles likely dominated Fe loss, with leaching as a secondary pathway. Ferrihydrite nanoparticles can persist as Si- and OM-coated colloids in floodplains, suggesting that nanoparticle leaching may have contributed to Fe export.^18^

In contrast to Fh-MCS, aggregates with OM addition showed a clear transformation of ferrihydrite to Fe­(II) carbonates, as evidenced by the decrease of Fe_Asc_ with simultaneous Fe_HCl_ increase. Previous work has also suggested the formation of OC-preserving carbonate-bound Fe­(II) species during the reductive dissolution of Fe­(III) oxides in paddy soils.^7^ As an electron donor and energy source, OC stimulates microbial Fe­(III) reduction, promoting ferrihydrite dissolution and siderite formation by local supersaturation.^14,42^ In addition, Fe­(III)-OM associations can have more structurally diverse and disordered morphologies than pure Fe­(III) minerals, making them more susceptible to reductive dissolution.^10,14^ However, the extent of reduction strongly depends on OM loading: at low to moderate OC/Fe ratios, OM can enhance Fe­(III) reduction by serving as an electron donor and increasing microbial activity, whereas at higher OM loadings, surface passivation and site blocking by OM coatings can inhibit reductive dissolution by limiting the access of microorganisms and reductants to reactive Fe sites.^10,42^ Notably, this transformation of ferrihydrite to Fe­(II) carbonates began already at 30 cm depth under intermittently oxic conditions, with episodic flooding enabling recurrent anoxia. Similar redox fluctuations in paddy soils promote the reductive dissolution of reactive Fe­(III) minerals and favor more crystalline OC-stabilizing Fe phases, depending on the mineral composition and leaching.^7,49^

In Py-MCS and Py-MOCS, synthetic pyrite partially transformed into weakly crystalline Fe­(III) oxides and oxalate-extractable Fe­(II/III) minerals via partial oxidation.^41^ The occurrence of amorphous FeS is relatively unlikely because reference minerals showed near-equal extraction of FeS in Fe_Asc_ and Fe_HCl_, yet Fe_HCl_ was largely absent. These phases were not resolved by Mössbauer spectroscopy, likely because of overlap with clay-Fe­(III), low Fe contents, and low abundance.^44,46^ The synthesized pyrite used here may be more reactive than natural pyrite, a known phenomenon,^41^ and likely enhanced in reactivity by lower crystallinity due to the inclusion of OM, clay, and sand during synthesis.^20^ This may explain the observed mineral transformations and high Fe losses in dissolved or colloidal form during pyrite oxidation.

Sd-MCS showed partial transformation into amorphous and crystalline Fe­(III) oxides, although a portion of siderite remained even in the topsoil. The persistence and formation of siderite at these depths were unexpected, given its redox sensitivity^50^ and redox fluctuations occurring in marsh soils favoring Fe­(III) oxide formation. Surface layer oxidation,^51,52^ passivation layer formation,^50^ and crystal lattice manganese (Mn) incorporation^51^ are processes that have been shown to successfully stabilize siderite against oxidation and are likely to occur in marsh environments. In Sd-MOCS, siderite was more stable, with only minor conversion to Fe­(III) phases. Siderite–organic acid associations are more stable than pure siderite,^50^ and dissolved organic reductants may further stabilize siderite by scavenging oxygen and modifying crystal growth by reducing surface energy differences.^53^

Instead, amorphous Fe­(III) oxides formed in the topsoil under dominating oxic conditions. In the permanently water-saturated zone (>45 cm soil depth), Fe­(II) minerals remained largely stable, regardless of mineral type. Along the redox gradient, our findings support our hypotheses that Fe­(III) oxides dominate in oxic zones, while both Fe­(II) mineral stabilization and formation—particularly siderite—increase with soil depth. However, partial Fe loss and redistribution indicate that these phases are at least partially dynamically cycled rather than persistently retained. This Fe­(II) mineral was already formed at intermediate depths (15–45 cm), despite oxic to suboxic conditions. Mössbauer data ruled out other carbonate minerals, such as ankerite or calcite, which would exhibit lower ΔE_Q_ values (∼1.40–1.65 mm s^–1^ at RT).^44^ Rothwell et al. recently made the observation that sorption of organic acids substantially slowed the oxidation of siderite in oxic environments, possibly due to stabilization by a surface passivation layer.^50^ Thus, once formed, siderite may persist through intermittent oxic phases.

Overall, the presence of OM significantly influenced Fe transformation dynamics across all experimental systems. In Fh-MOCS, OM addition promoted the reductive dissolution of ferrihydrite by serving as both an electron donor and a carbon source, stimulating microbial Fe­(III) reduction and the subsequent formation of Fe­(II) carbonates. In Sd-MOCS, OM enhanced siderite stability under oxic conditions, likely through organic-ligand-induced passivation and oxygen scavenging by dissolved organic reductants, allowing siderite to persist in periodically oxic horizons. In Py-MOCS, the inclusion of OM during synthesis likely increased pyrite reactivity and susceptibility to oxidation. Thus, OM functions in a dual role, accelerating Fe redox cycling and stabilizing specific Fe­(II) minerals through complexation and surface modification. Overall, OM acts as a key regulator of Fe phase transformation, capable of either promoting Fe­(III) reduction or inhibiting oxidation, thereby controlling the balance between Fe­(III) and Fe­(II) mineral stability along the marsh redox gradient.

### 3.3 Association of OC with Reactive Fe­(III) and Fe­(II) Phases

After 6 months of in situ incubation, the original OC contents in Fh-MOCS decreased with depth, ranging from 8.7 ± 2.9 mg kg^–1^ at 15 cm to 6.2 ± 0.8 mg kg^–1^ at 60 cm (Figure 8a). For Sd-MOCS and Py-MOCS, OC peaked at 30 cm (8.2 ± 3.6 mg kg^–1^ and 10.7 ± 6.7 mg kg^–1^, respectively) and declined toward the bottom layer (5.6 ± 1.4 mg kg^–1^ and 4.8 ± 0.3 mg kg^–1^ at 60 cm) at the end of the incubation. Compared to preincubation levels (Figure 8a), the average retention of the originally sorbed OC ranged from 46.1–72.2% for Fh-MOCS, 39.3–61.7% for Sd-MOCS, and 38.6–85.4% for Py-MOCS. Although OC contents declined overall with depth, Fe­(II) minerals supported OC retention under reduced conditions. Organic C concentrations in the inner and outer rings of Sd-MOCS and Py-MOCS sometimes exceeded both the previously measured OC concentration of the soil at the particular depth and the core preincubation levels (Figure 8a).

**Figure 8:**
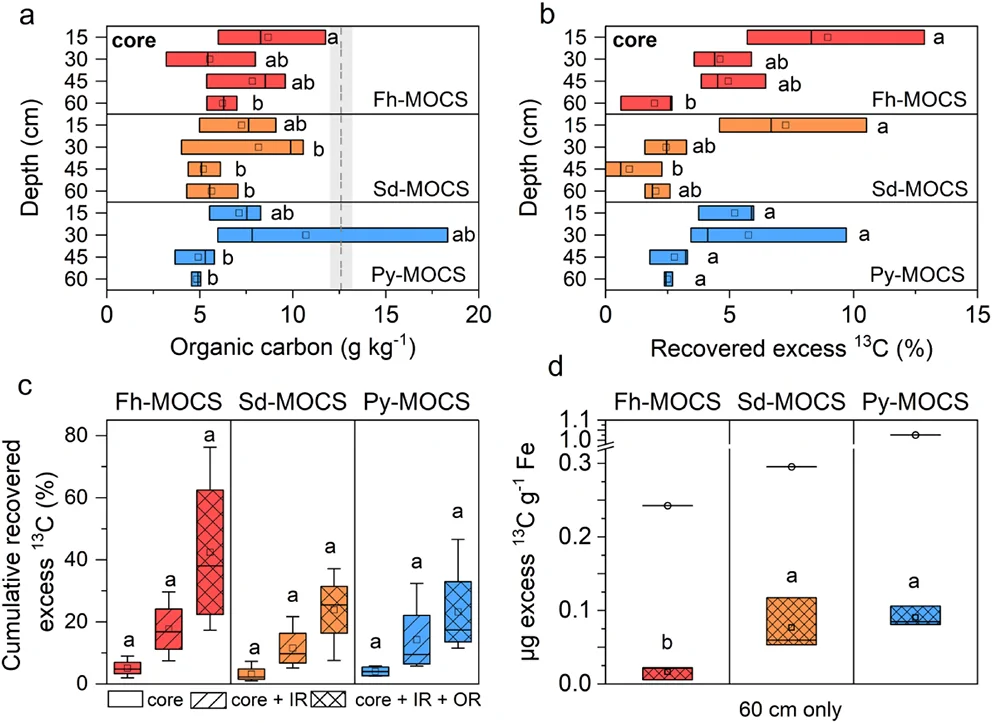
Postincubation analysis of ferrihydrite-based organic clay–sand aggregates (Fh-MOCS), siderite-based MOCS (Sd-MOCS), and pyrite-based MOCS (Py-MOCS). (a) Total OC in aggregate cores with the gray bar indicating preincubation levels; (b) excess ^13^C (% vs preincubation) in cores; (c) cumulative ^13^C recovery across sampling zones, including inner (IR) and outer (OR) rings; (d) recovered ^13^C tracer in cores under reducing conditions, normalized to Fe content, with initial values shown for comparison.

Excess ^13^C in aggregate cores showed similar trends to total OC in relative ^13^C tracer recovery after 6 months (Figure 8b). For Fh-MOCS, ^13^C recovery at the termination of the experiment decreased from 9.0 ± 3.62% at 15 cm to 1.97 ± 1.17% at 60 cm. Sd-MOCS- and Py-MOCS followed the same trend, with the lowest values at 45 cm (0.96 ± 1.18% for Sd-MOCS) and slightly higher retention at 60 cm (2.03 ± 0.52% and 2.5 ± 0.7%, respectively). These results indicate that Fe­(II) minerals in membrane cylinders buried in saturated soil layers preserved more sorbed ^13^C than ferrihydrite.

When calculated as total cumulative ^13^C recovery, ferrihydrite aggregates showed the highest values in the topsoil across the entire sampling zones (core, inner ring, outer ring), especially in the outer ring (up to ∼75% total ^13^C recovery; Figure 8). However, with soil depth, ^13^C recovery sharply declined. In contrast, in Sd-MOCS and Py-MOCS, ^13^C tracer was more widely scattered across all sampling zones at all depths, especially in water-saturated layers. Notably, in some outer ring zones of Py-MOCS and Sd-MOCS (Sd-MOCS 30 and 45 cm, Py-MOCS 60 cm), more excess ^13^C was recovered than in the cores, suggesting substantial lateral redistribution of ^13^C-labeled OM into surrounding soil (Figure 8), suggesting its desorption and resorption.

ANOVA analysis (Table 3) revealed that Fe mineral type markedly affected ^13^C retention but not total OC content. Soil depth had a significant effect on both OC and excess ^13^C, confirming the importance of redox stratification. An interaction between Fe mineral type and soil depth was also significant for OC but not for excess ^13^C, as was the interaction between Fe mineral type and the sampling zone of the ring.

**Table 3:** Two-Way ANOVA Parameters of Organic Carbon and Excess ^13^C (* *p* < 0.05, ** *p* < 0.01, *** *p* < 0.001) for Fe Mineral, Depth, and Sampling Zone (Core, Inner Ring, Outer Ring)

	Organic carbon		Excess ^13^C	
ANOVA	*F*	*p*	*F*	*p*
Fe mineral	0.83	0.44	11.81	***
Depth	107.66	***	21.16	***
Zone	96.48	***	12.15	***
Fe mineral × depth	4.43	***	1.50	0.19
Fe mineral × zone	1.22	0.31	2.55	*
depth × zone	23.38	***	0.47	0.83

Fe­(II) mineral-MOCS partially preserved added particulate reed-derived OC during the six-month incubation under the highly dynamic field conditions occurring in tidal marshes. For total OC in MOCS, the depth is most decisive (*p* < 0.001), with some interaction between Fe minerals and depth (Table 3). The incubated aggregates further showed that ^13^C-labeled reed DOM sorbed onto synthetic aggregates (Fe-MOCS) consisting of typical soil constituents (Fe minerals, OM, clay, sand) was at least partially preserved. During incubation, reed-derived particulate organic matter (POM) contributed to Fe mineral–organic complex formation through multiple pathways. These may include (i) the partial solubilization of reed litter into DOM enriched in carboxyl- and phenol-rich compounds, which can bind directly to Fe oxide surfaces via ligand exchange; (ii) microbial processing of litter into biomass and subsequent necromass deposition, where reactive mineral surfaces selectively retain plant- and microbial-derived compounds; and (iii) the remobilization of sorbed OC and microbial recycling thereof.^54–56^ As observed in Fh-MOCS under oxic conditions, POM-derived total OC and sorbed ^13^C-DOM were largely retained in the upper oxic zones. Association with poorly crystalline Fe­(III) oxides (Figures 4 and 5) indicates classical sorptive stabilization. This could have involved (i) existing Fe­(III) oxides,^1^ (ii) Fe­(III) oxides formed by Fe­(II) oxidation,^57^ and (iii) organics occupying functional sites and formation of an oxide passivation layer protecting Fe­(II)-OM.^50^

However, Fe-OC associations at greater soil depths, which are more exposed to groundwater table fluctuations and anoxic conditions, were also at least partially stabilized by Fe­(II) mineral phases (60 cm, 39–51% of total OC, 2–2.5% of sorbed ^13^C recovered in cores). For the stabilization of mineral-sorbed OC, as quantified by excess ^13^C, both Fe minerals and depth had a significant impact (*p* < 0.001). The inverse distribution of ferrihydrite (Fe_Asc_) and siderite (Fe_HCl_), as identified by sequential extraction and Mössbauer spectroscopy, demonstrates distinct depth-dependent Fe mineral trends. The preservation of the ^13^C tracer along this gradient indicates that OC stabilization can occur via both Fe­(III) oxides and Fe­(II) minerals under fluctuating redox conditions in tidal marsh soils. Tracer recovery in cores was comparable for Fe­(III)- and Fe­(II)-dominated systems (1.4–28.5% vs 2–25%), confirming our hypotheses 2 and 3, though the latter was, under the conditions of the field experiment, limited to siderite.

The interaction of Fe mineral and the zone of the membrane cylinders was also significant (*p* < 0.05), suggesting that tracer redistribution patterns vary with mineral type and distance from the aggregate core. Thus, depth and sampling zone are dominant factors shaping both the OC and ^13^C patterns. Mineral identity specifically controls ^13^C preservation, likely through differences in redox stability, surface chemistry, or OM binding mechanisms.

In Fh-MOCS, OM enhanced siderite formation, serving as both a C source and an electron donor, which accelerated Fe­(III) reduction and ferrihydrite dissolution.^5^ This mutually reinforcing process challenges the Fe­(III) oxides’ role in OC stabilization and mobility under suboxic or anoxic conditions and is consistent with observations from gleyic forest soils, where reductive dissolution of Fe­(III) oxides increased dissolved organic carbon (DOC) mobility by removing mineral sorption sites, highlighting the dynamic and reversible nature of Fe–OC associations under fluctuating redox conditions.^58^

In paddy soils, secondary carbonate-bound Fe­(II) species have been suggested to contribute to OC preservation during redox-fluctuation-induced Fe­(III) oxide dissolution.^7^ There, limited Fe­(II) mineral transformation in the lower soil depth favors the retention of sorbed OC.^50,57^ Passivation in oxic upper layers through oxidative layer formation, e.g., of siderite, is another possibility for stabilizing Fe­(II) mineral-OM associations, thus potentially enhancing OM occlusion.^50^

The high ^13^C tracer concentration in OR zones (especially for Fh-MOCS) indicates that although sorbed OM is stabilized by association with Fe­(III) oxides, substantial OM transport occurred. If this transport had primarily resulted from reductive Fe­(III) dissolution and OM desorption, enhanced mineralization and a greater loss of the ^13^C tracer would be expected. The comparatively high tracer recovery, therefore, suggests that this process was not the dominant driver of OM transport in the OR zones. Instead, OM mobilization and transport to the OR zones are more consistent with the disintegration of the Fh-MOCS into smaller, mobile colloids, including Fe mineral-OM or Fe mineral-OM-clay complexes. Such disintegration may be induced by OM coating, vertical flow,^48^ or mineral transformation.^59^ Thomas-Arrigo and Kretzschmar reported a mobilization of OM as organic-Fe colloids after a pH increase caused by microbial reduction of SRO Fe mineral phases under anoxic conditions, with colloids remaining in suspension even after reoxidation.^60^ Consistent with this interpretation, Kubeneck et al. demonstrated in Elbe intertidal flat sediments that reductive ferrihydrite dissolution can lead to diffusive or advective Fe loss from mesh bags, with the extent of mineral export strongly controlled by sediment texture.^11^ Colloid and Fe-OM mobility are physically constrained in finer-grained soils.^48^

Thus, Fh-MOCS contributed to OC stabilization in oxic topsoil but also facilitated OM mobility in hydromorphic zones. In contrast, Fe­(II)-MOCS showed moderate persistence of mineral-sorbed OC at the lowest depth, primarily in the core, suggesting less diffusion outward, but also higher microbial decomposition. Ferrihydrite and FeS strongly sorb and selectively retain aromatic, oxygen-rich DOM, while pyrite exhibits lower sorption.^21^ Under a shift from oxic to reducing conditions in an aquifer, OM-bearing biogenic ferrihydrite transformed into Fe carbonates and sulfides, leading to substantial changes in the composition and preservation of associated OM.^61^ FeS can act as a secondary sink for OM, either by almost completely reimmobilizing the DOM released during ferrihydrite sulfidation or by retaining OM within FeS-OM coprecipitates.^62^ Siderite is stable under anoxic conditions, enabling long-term preservation of associated OM, while organic ligands can further enhance stability even during oxic shifts by forming Fe–organic surface layers.^50^

These differences highlight that Fe mineral identity not only governs OM binding strength but also spatial retention dynamics across the redox-active soil microenvironments. The observed loss of ^13^C from the core filled with Fh-MOCS and its accumulation in the inner and outer rings did not scale proportionally with Fe loss (Figure S2). Excess ^13^C losses from the core exceeded Fe losses (Figure S2). Fh-MOCS lost about one-third of its total iron (29.3–36.1% Fe), Sd-MOCS the least (0–27.4% Fe), and Py-MOCS the most (47.9–76.9% Fe), yet only 1–9% of the originally mineral-sorbed OM persisted in cores. This indicates that OC preservation was in large parts decoupled from the stability of the initial Fe phase. Possible interpretations are (i) OC destabilization through Fe­(III) dissolution followed by reprecipitation of Fe­(III) oxides, enhancing OM retention in surrounding soil,^7^ or (ii) desorption of OC due to reduced sorption capacity under saline tidal conditions,^63^ with secondary retention by native soil minerals, especially in finer-textured upper horizons.^5^ Similarly, the sporadic rise of OC concentrations in the inner and outer rings of Sd-MOCS and Py-MOCS (Figure 8a,c), sometimes exceeding both the baseline OC concentration of the soil at the respective depth and the core preincubation levels, suggests net OC inputs or secondary retention processes in the surrounding soil. Such increases could result from vertical input of OC from aboveground vegetation detritus during the postsummer season.^64^ Another possibility is enhanced retention by newly formed Fe­(III) phases that migrated from the membrane cylinders into surrounding soil during Fe oxidation, increasing local binding capacity.^57^ The absence of this trend in Fh-MOCS, however, suggests Fe­(II)-specific dynamics. Thus, it may reflect Fe­(II) oxidation or possibly weaker adherence of OM or clay-OM in Fe­(II) minerals-MOCS, potentially due to the point of zero charge or preferential molecular fractionation of OM.^21^ Alternatively, redox fluctuations could induce the formation of FeS or FeS_*x*_ colloids by oxidation and resulfidation of produced Fe­(III) oxides.^59^

Overall, the disproportionate loss of excess ^13^C relative to Fe, together with substantial tracer accumulation in the surrounding soil, indicates that Fe redox cycling with secondary OC retention was the dominant process. In the examined brackish marsh subject to episodic flooding, Fe­(III) dissolution followed by reprecipitation and sorptive retention in adjacent soil, therefore, appears more significant than salinity-driven desorption, while Fe­(II)-specific processes and FeS or FeS_*x*_ colloid formation likely played secondary roles under fluctuating redox conditions.

Not only does OM drive redox shifts, but it also alters the trajectory of Fe phase transformation. The presence of OM transformed, for instance, ferrihydrite into siderite in Fh-MOCS, shifting the mode of OC association from Fe­(III)-dominated surface sorption to stabilization by secondary Fe­(II) minerals. Efficient resequestration of DOC can occur via coprecipitation with FeS during release by Fh-OC coprecipitate sulfidation.^62^ Siderite formation has been shown to occur during microbial reduction of OM-ferrihydrite complexes by *Geobacter bremensis* depending on OM loading, but was retarded at higher loadings due to Fe^2+^ complexation and surface site blocking.^42^ Similarly, Cismasu et al. observed secondary Fe­(II) mineral formation after reductive dissolution of OM-bearing ferrihydrite; ∼30% of the initial OC was redistributed to newly formed Fe phases or separated as OM globules.^61^ Total OC and inorganic C increased during their open-system incubation. Overall, both preserved and newly formed Fe minerals—including ferrihydrite, siderite, pyrite—contributed to OC stabilization and cycling. The relative importance of these phases depends on redox conditions and OM availability. Fe­(III) oxides were most effective in preserving OC in oxic horizons. In contrast, siderite and pyrite showed comparable sorbed ^13^C tracer preservation in deeper, water-saturated zones, where Fe­(II)-dominated phases remained stable, indicating a direct role of Fe­(II) minerals in OC stabilization.

## Supplementary Material



## Notes

Soil characterization methods; mineral synthesis and characterization; aggregate preparation and transport details; excess ^13^C calculations; reference mineral extraction efficiencies; pre- and postincubation Fe and ^13^C recovery data; Fe and excess ^13^C loss relationships; fitted Mössbauer spectra and site populations (PDF)
