# Supplementary material for: Fe(III)-(II) Mineral Transformation and Associated Organic Matter Stabilization Processes in a Tidal Marsh Soil
Source: Environ Sci Technol. 2026 Aug 3;60(32):22530–41. doi: 10.1021/acs.est.6c05347 (PMC13492270; doi:10.1021/acs.est.6c05347)
Supplement: Supplementary file 1 [file es6c05347_si_001.pdf]

Supporting Information for

Fe(III)-(II) Mineral Transformation and Associated Organic Matter Stabilization Processes in a Tidal Marsh Soil

Jan Jagode[a,], Jannis F. Carstens[b], Hamed Kashi[c], Dörthe Holthusen[d], Kevin Tran[e], Heiner Fleige[c], Jana Carus[f], Elmar Fuchs[d], Franz Renz[e], Sandra Spielvogel[c], Georg Guggenberger[a]*

*Corresponding Author: Jan Jagode, Email: jagode@ifbk.uni-hannover.de

[a]Section Soil Science, Institute of Earth System Sciences, Leibniz University Hannover, Herrenhäuser Str. 2, 30419 Hannover, Germany

[b]Technische Universität Clausthal, Adolf-Roemer-Str. 2A, 38673 Clausthal-Zellerfeld, Germany

[c]Institute for Plant Nutrition and Soil Science, Christian-Albrechts-University Kiel, Hermann-Rodewald-Str. 2, Kiel, 24118, Germany

[d]Department Ecological Interactions, Federal Institute of Hydrology, Am Mainzer Tor 1, 56068 Koblenz, Germany

[e]Institute of Coordination Chemistry, Leibniz University Hannover, Callinstraße 3-9, 30419 Hannover, Germany

[f]Department of Plant Ecology, Institute of Ecology, Technical University of Berlin, Rothenburgstraße 12, 12165 Berlin, Germany

Number of Pages: 20

Number of Texts: 6

Number of Figures 9

Number of Tables: 5

Texts:

Text S1. Sampling & characterization details

Text S2. Mineral synthesis confirmation - XRD spectra method details

Text S3. Quartz sand and illite preparation for synthetic aggregates

Text S4. Preparation & transport of membrane incubation cylinders

Text S5. Isotopic baseline of pre-incubation materials & post-incubation recovery

Text S6. Porewater extraction and analysis

Figures:

Figure S1a. XRD spectrum of synthesized siderite (green lines) without sand and clay addition.

Figure 1b. XRD of synthesized pyrite without sand & clay addition. Pyrite (green lines), the remaining reflexes belong to pre-wash sulfur impurities (<5%).

Figure S1c. XRD of synthesized ferrihydrite without sand & clay addition. Characteristic two broad reflexes around 34° and 63° degrees.

Figure S2. Relationship between Fe mineral stability and redistribution of ^{13}C-labeled organic carbon across the core, inner ring, and outer ring of mineral–organic clay sands (MOCS). Recovered excess ^{13}C is shown as a function of Fe loss from the core for the core (top left), inner ring (top right), and outer ring (bottom left), and as a function of Fe recovery as the original mineral phase in the core (bottom right). Symbols denote incubation depth and colors indicate Fe mineral type (Fh: ferrihydrite, Sd: siderite, Py: pyrite).

Figure S3. Pourbaix diagram for pore water composition of lowest examined depth at 60 cm. Thermodynamical more stable Fe minerals were suppressed to show stability of employed ferrihydrite, siderite and pyrite. Created with Geochemist's workbench, diagram conditions: Fe(OH)3(ppd), T = 25 °C, P = 1 bars, a[main] = $10^{-2.398}$, a[H_2O] = 1, a[Cl^-] = 1, a[SO_4^{2-}] = $10^{-2.703}$, a[Ca^{2+}] = $10^{-2.498}$, a[Na^+] = $10^{-1.773}$, a[K^+] = $10^{-3.824}$, a[Mg^{2+}] = $10^{-2.955}$, a[Mn^{2+}] = 1, a[HCO_3^-] = $10^{-2.745}$; Suppressed: Fe(CH3COO)2+, Fe(CH3COO)3-, Ferrite-2-Ca, Ferrite-Ca, Ferrite-Cu, Ferrite-Mg, Ferrite-Zn, Goethite, Hematite, Pyrrhotite, Troilite

Figure S4. Left: Empty membrane cylinders. Right: Filled membrane cylinders

Figure S5a. Krautsand high marsh profile at the beginning of the field incubation. Filled membrane cylinders are inserted horizontally at 15, 30, 45 and 60 cm depths.

Figure S5b. Krautsand high marsh profile at the end of the field incubation.

Figure S6. Fitted room-temperature Mössbauer spectra of recovered Fe mineral–organic–clay–sand aggregates after six months of in situ incubation in tidal marsh soil.

Tables:

Table S1. Isotopic composition and organic carbon content of pre-incubation materials used in the field experiment. Fe-MOCS refers to artificial Fe mineral-organic matter-clay-sand aggregates ferrihydrite (Fh), siderite (Sd) and and pyrite (Py). POM indicates added particulate organic matter (ground reed).

Table S2. Mössbauer spectroscopy parameters (Isomer shift δ and quadrupole splitting ΔEQ, in mm s^{-1}) of Fe mineral-organic-clay-sand of ferrihydrite, siderite and pyrite (Fh-MOCS, Sd-MOCS, Py-MOCS) incubated with organic carbon at different soil depths compared to literature (Murad 1988; Murad und Cashion 2004; Gil et al. 1992). Uncertainty is given in parentheses. *Manually fitted.

Table S4. Mössbauer spectroscopy parameters: Isomer shift δ, quadrupole splitting ΔE_Q and site population of Fe mineral-organic-clay-sand of ferrihydrite, siderite and pyrite (Fh-MOCS, Sd-MOCS, Py-MOCS). Uncertainty is given in parentheses. *Manually fitted due to parameter overlap.

Table S5. Test of Fe extraction efficiency by sequential extraction according to Lenstra et al. (2019; 2021). MLR = Mineralmühle Leun, Rau Gmbh (Leun, Germany), LAN = LANXESS Deutschland GmbH (Köln, Germany), syn. = synthesized

Table S6. Concentrations of dissolved constituents in soil porewater (60 cm depth) extracted by centrifugation.

Text S1. Sampling & characterization details

The Krautsand high marsh site was characterized by mesophilic grassland vegetation, dominated by sweet grasses (Arrhenatherum elatius, Festuca rubra, Anthoxanthum odoratum, Cynosurus cristatus, also found: Daucus carota, Plantago lanceolata, Ranunculus acris, Rumex acetosa, Ajuga reptans).

Disturbed soil samples were collected from the soil profile horizon to the groundwater table. Samples from reduced horizons for Fe speciation were flushed with N_2 and stored airtight in PE bags. For oxygen insensitive analyses, soil samples were dried at 30–35°C in a ventilated oven for 7 days, crushed manually and sieved to <2 mm, visible plant or root material was removed. Soil pH and electrical conductivity (EC) were measured in 0.01 M $CaCl_2$ and deionized water, respectively, at a 1:2.5 (w/w) soil-to-solution ratio.[27] Soil texture was determined via sieving and sedimentation after OM destruction by H_2O_2 (30%) addition and carbonate dissolution by HCl.[27] Calcium carbonate content was measured using Scheibler's volumetric gas method.[27] Organic C contents (% dry weight) and $^{12}C/^{13}C$ stable-isotope ratios were measured by total combustion of acid-treated samples by elemental analysis-isotope ratio mass spectrometry (EA-IRMS; Elementar vario ISOTOPE cube coupled to an Elementar isoprime precisION mass spectrometer, Langenselbold, Germany). Isotopic values are expressed in $\delta^{13}C$ relative to the Vienna Pee Dee Belemnite (VPDB) standard. For dithionite extractable Fe (Fe_d), 0.5 g dried soil were extracted with 40 ml 0.68 M Na citrate solution and 0.4 g freshly added Na dithionite and shaken for 16 h.[28] To obtain oxalate extractable Fe (Fe_o), 1 g sample was added to a solution of 0.2 M NH_4 oxalate and 0.2 M oxalic acid at pH 3 and shaken for 4 h in the dark.[29] Extracted Fe was determined by ICP-OES (Varian 725-ES, Varian Inc., Palo Alto, USA) for both extractions with matrix-adjusted calibrations.

Text S2. Mineral synthesis confirmation - XRD spectra method details

Synthesized mineral purity was tested without sand and clay additions and identified as the targeted phase. Mineralogical composition of Fe mineral phases was analyzed by X-ray diffraction (XRD) using a Siemens D500 diffractometer (Siemens AG / Bruker AXS GmbH, Karlsruhe, Germany). Measurements were performed using Cu K_α radiation (λ = 1.5406 Å) operated at 40 kV and 30 mA. Samples were analyzed over a 2θ range of 5–70° with a step size of 0.02° and a counting time of 1–2 s per step. Diffractograms were evaluated using standard reference patterns, and mineral phases were identified based on their characteristic reflections.

Text S3. Quartz sand and illite preparation for synthetic aggregates

Quartz sand G0-03T (Schlingmeier, Germany) was washed in 32% HCl for 48 h and occasionally stirred manually. Afterwards, it was intensively rinsed with deionized water until EC < 10 µS cm^{-1}. Muscovite-type mica Mica SFG70 (Aspanger, Austria) was used. The clay fraction < 2 µm, henceforth called illite based on parent material and grain size, was separated via sedimentation, precipitated through $CaCl_2 \cdot 2H_2O$ addition, washed with deionized water, centrifuged and decanted until EC < 10 µS cm^{-1}. The gained illite was shock frozen via dropwise addition to liquid N_2 and freeze dried.

Text S4. Preparation, transport & retrieval of membrane incubation cylinders

50 ml polypropylene sample tubs (ROTILABO®, Carl Roth, Karlsruhe, Germany) were modified via cutting out two wall segments (4x4 cm). A polyamide mesh (15 µm mesh with, PA-15/10, Franz Eckert GmbH, Waldkirch, Germany) was fixed around the tubes with ACRIFIX® 1R0192 glue (nattmann GmbH, Willich, Germany). The membrane cylinders were then filled with 3 layers (inert quartz sand–45 g Fe-MCS or 45 g Fe-MOCS–inert quartz sand), so that only Fe mineral aggregates (Table 2) were in contact with the membrane. After the membrane cylinders were filled with the samples, they were shock frozen via immersion in liquid N_2 and separately packed in freezer bags. Until transport to the field site, the freezer bags were stored in airtight wide-neck drums in a cold room under inert gas. While transporting, the sample bags filled drums were refrigerated with dry ice, thus ensuring an oxygen-free atmosphere.

After retrieval, samples were kept at 4 °C to slow biological activity prior to processing. To minimize atmospheric oxygen exposure during transport, membrane cylinders were recovered together with the surrounding field-moist soil, sealed in PP containers, and additionally enclosed in freezer bags. The cylinder fillings therefore remained embedded in moist soil until processing. During processing, the membrane-cylinder fillings were immediately shock-frozen in liquid N_2, exposed surfaces discarded; sectioned with cleaned tools, and stored under N_2 atmosphere until analysis. Short-term oxygen exposure during excavation and handling cannot be fully excluded, but direct exposure of the incubated aggregates to air was minimized before freezing and anoxic storage.

Text S5. Isotopic baseline of pre-incubation materials & post-incubation recovery

The isotopic and carbon characteristics of all materials were determined prior to field deployment to establish reference values for subsequent tracer calculations. These baseline data were used to quantify ^{13}C enrichment, calculate excess ^{13}C, and assess organic carbon redistribution during the incubation.

Stable carbon isotope ratios ($\delta^{13}C$) and organic carbon contents were determined using an Elementar vario ISOTOPE cube elemental analyzer coupled to an Elementar isoprime precisION isotope ratio mass spectrometer (Elementar Analysensysteme GmbH, Langenselbold, Germany). Isotope ratios are reported relative to VPDB. Samples were decarbonated by addition of 10% HCl and/or HCl fumigation.

Sample details:

- ^{13}C-tracer–labeled reed: Ground Phragmites australis biomass enriched in ^{13}C, used as a source of labeled dissolved organic matter (DOM) in combination with unlabeled particulate organic matter (POM).
- ^{13}C-tracer–labeled Fh-MOCS: Artificial ferrihydrite-OM-clay-sand aggregates synthesized using ^{13}C-enriched organic matter, without added POM.
- ^{13}C-tracer–labeled Fh-MOCS + reed POM: Ferrihydrite-based MOCS synthesized with ^{13}C-labeled OM and amended with unlabeled reed POM prior to incubation.
- ^{13}C-tracer–labeled Py-MOCS: Artificial pyrite-OM-clay–-sand aggregates synthesized using ^{13}C-enriched organic matter, without added POM.
- ^{13}C-tracer–labeled Py-MOCS + reed POM: Pyrite-based MOCS synthesized with ^{13}C-labeled OM and amended with unlabeled reed POM prior to incubation.
- ^{13}C-tracer-labeled Sd-MOCS + reed POM: Siderite-based MOCS synthesized with ^{13}C-labeled OM and amended with unlabeled reed POM prior to incubation

Text S6. Porewater extraction and analysis

Soil porewater was obtained by centrifugation of field-moist soil samples using pre-cleaned polypropylene centrifuge tubes. Samples were centrifuged at 5000 g for at least 30 min (Zentrifuge ROTANTA 460, Andreas Hettich GmbH & Co. KG, Tuttlingen, Germany), and the supernatant was immediately decanted, filtered through 0.45 µm cellulose acetate filters, and stored at 4 °C until analysis. Major anions and cations were quantified by ion chromatography (Dionex ICS-90, Dionex/Thermo Fisher Scientific, Sunnyvale, CA, USA). Calibration was performed with multi-ion standards, and analytical quality was ensured through the inclusion of blanks, replicate samples, and certified reference solutions. Bicarbonate was analyzed by titration with 0.1 M HCl and methyl orange as indicator. Based on measured porewater chemistry, thermodynamic stability fields of Fe phases were evaluated using Pourbaix diagrams generated with Geochemist's Workbench.

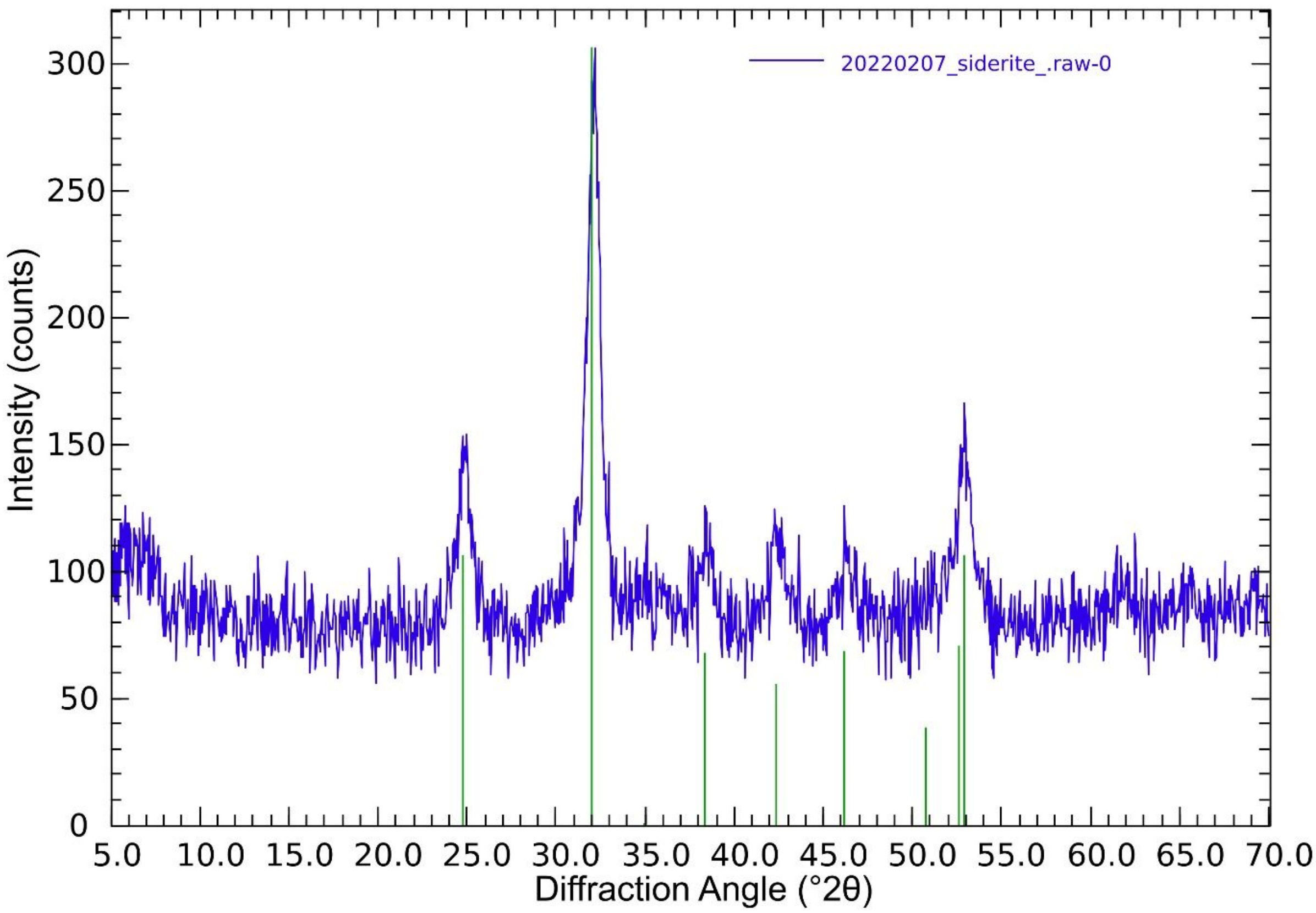

Figure S1a. XRD spectrum of synthesized siderite (green lines) without sand and clay addition.

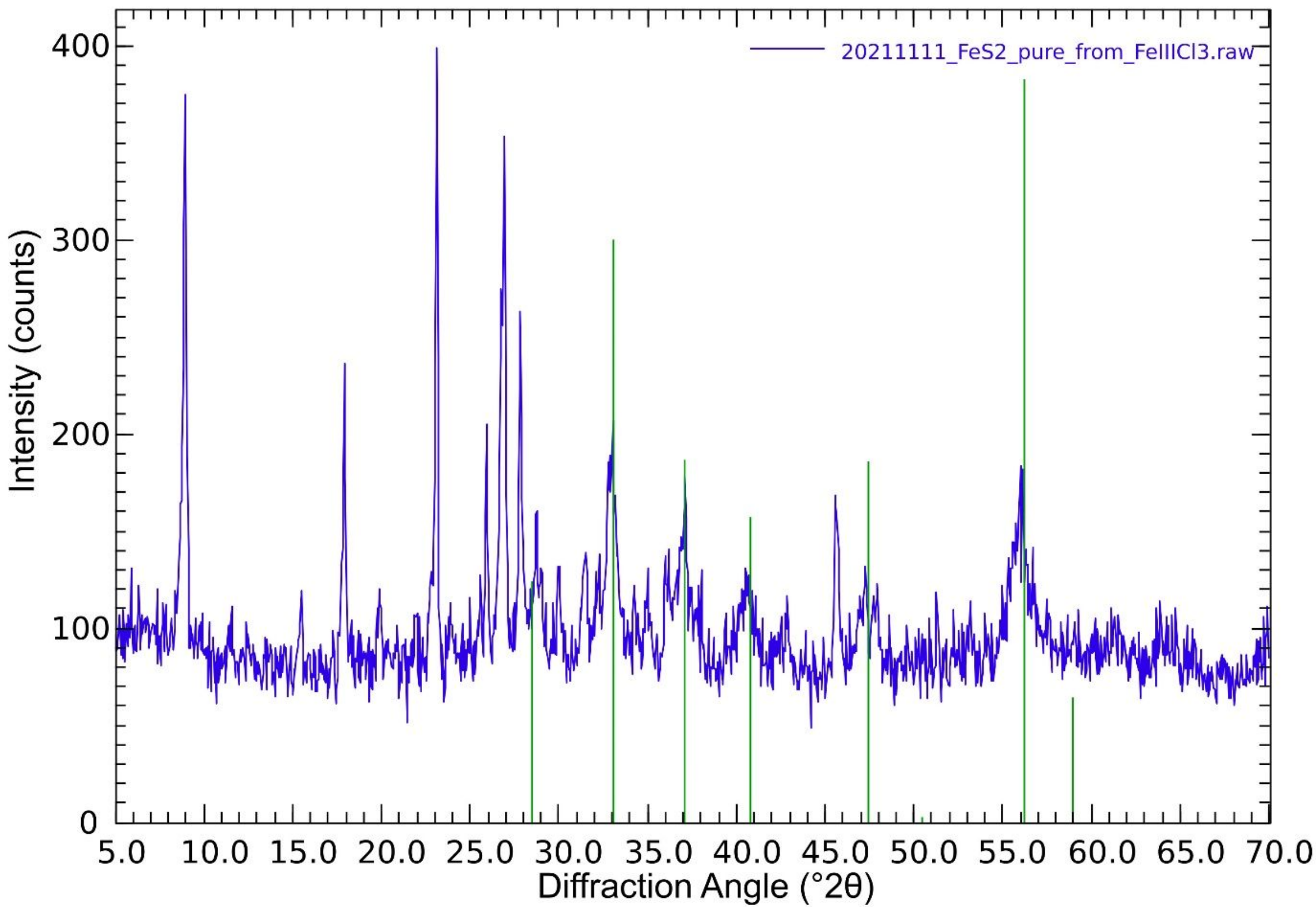

Figure 1b. XRD of synthesized pyrite without sand & clay addition. Pyrite (green lines), the remaining reflexes belong to pre-wash sulfur impurities (<5%).

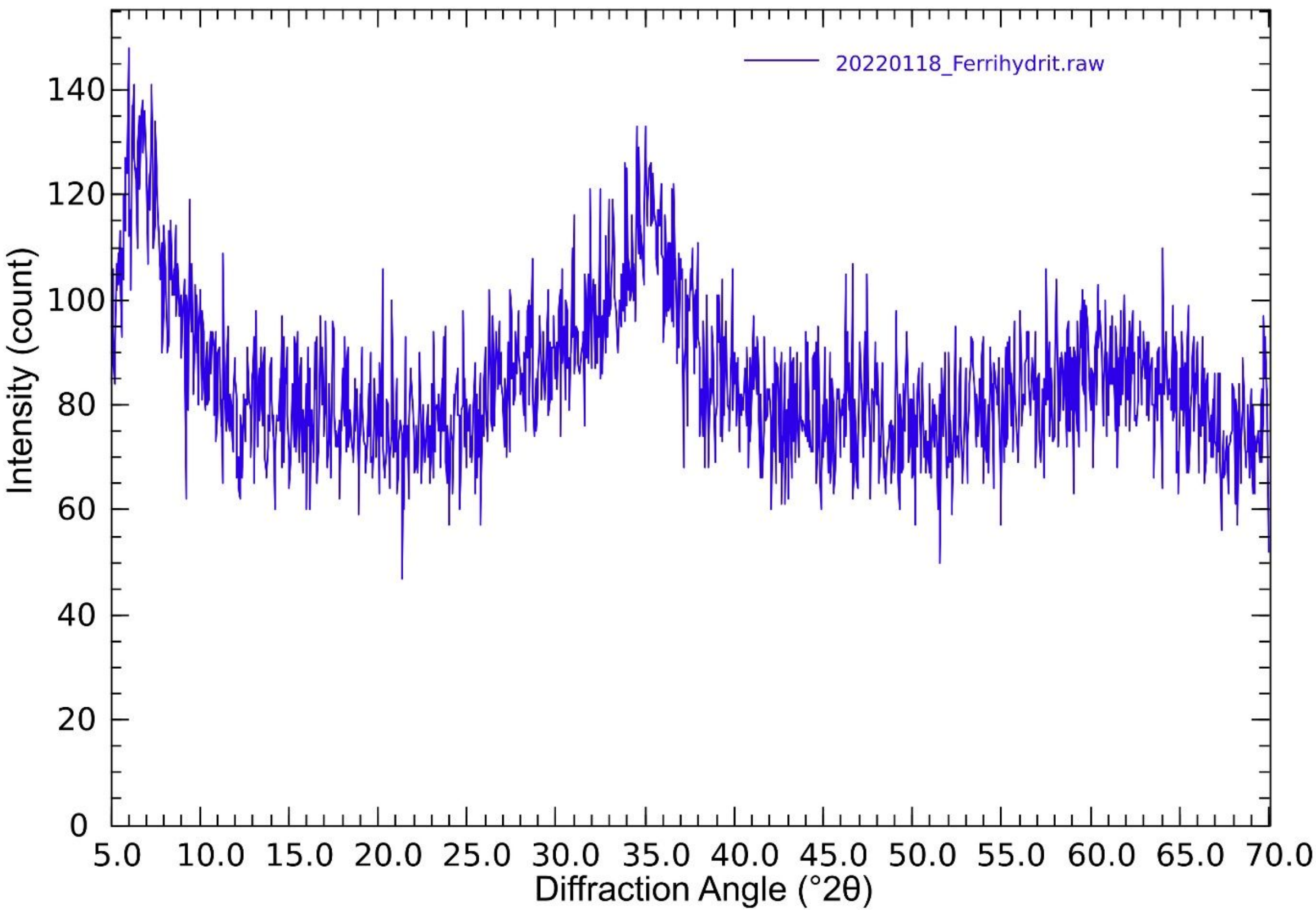

Figure S1c. XRD of synthesized ferrihydrite without sand & clay addition. Characteristic two broad reflexes around 34° and 63° degrees.

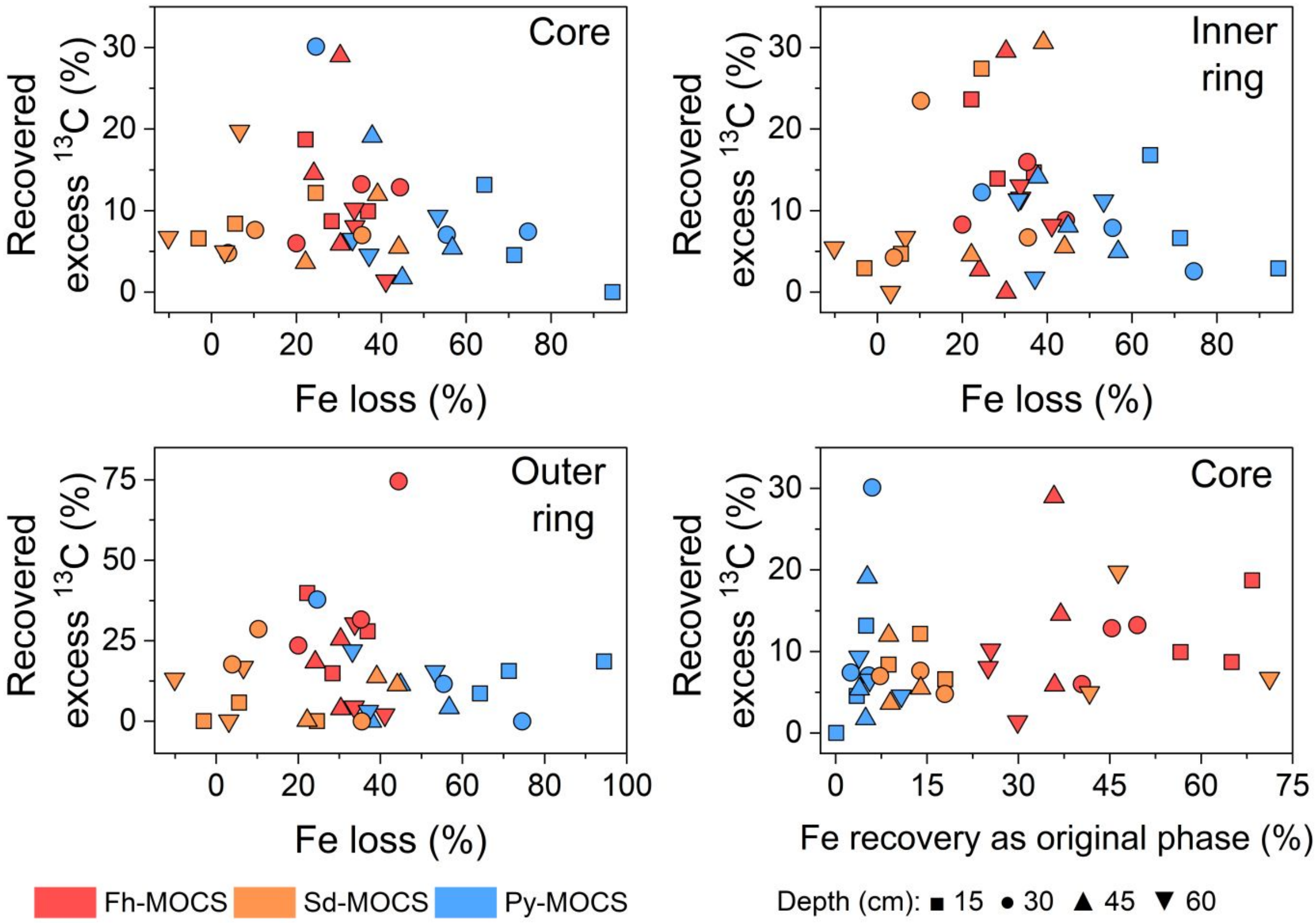

Figure S2. Relationship between Fe mineral stability and redistribution of ^{13}C-labeled organic carbon across the core, inner ring, and outer ring of mineral–organic clay sands (MOCS). Recovered excess ^{13}C is shown as a function of Fe loss from the core for the core (top left), inner ring (top right), and outer ring (bottom left), and as a function of Fe recovery as the original mineral phase in the core (bottom right). Symbols denote incubation depth and colors indicate Fe mineral type (Fh: ferrihydrite, Sd: siderite, Py: pyrite).

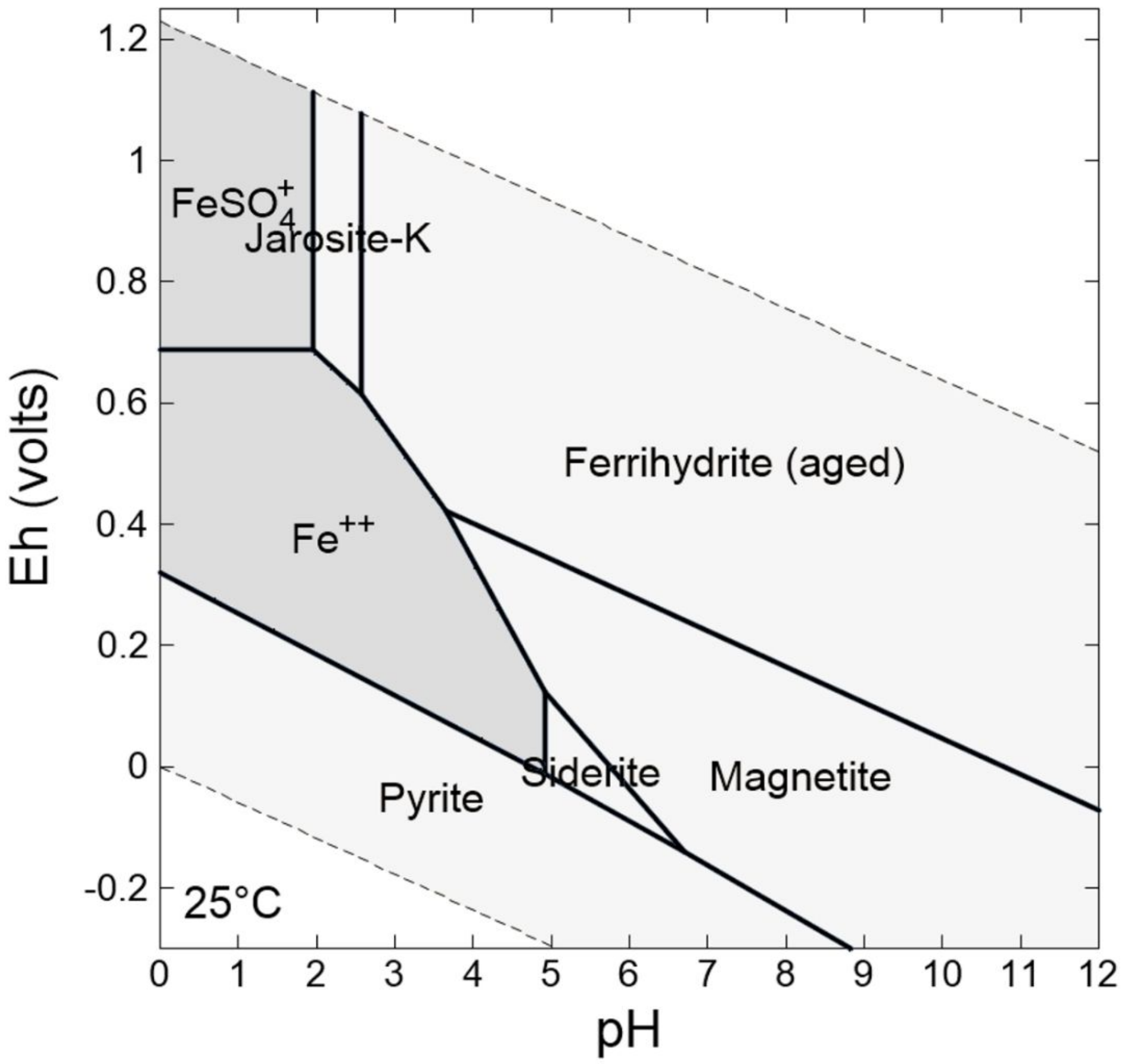

Figure S3. Pourbaix diagram for pore water composition of lowest examined depth at 60 cm. Thermodynamical more stable Fe minerals were suppressed to show stability of employed ferrihydrite, siderite and pyrite. Created with Geochemist's workbench, diagram conditions: Fe(OH)3(ppd), T = 25 °C, P = 1 bars, a[main] = 10-2.398, a[H_2O] = 1, a[Cl^-] = 1, a[SO_4^{2-}] = 10-2.703, a[Ca^{2+}] = 10-2.498, a[Na^+] = 10-1.773, a[K^+] = 10-3.824, a[Mg^{2+}] = 10-2.955, a[Mn^{2+}] = 1, a[HCO3-] = 10-2.745; Suppressed: Fe(CH3COO)2+, Fe(CH3COO) 3-, Ferrite-2-Ca, Ferrite-Ca, Ferrite-Cu, Ferrite-Mg, Ferrite-Zn, Goethite, Hematite, Pyrrhotite, Troilite

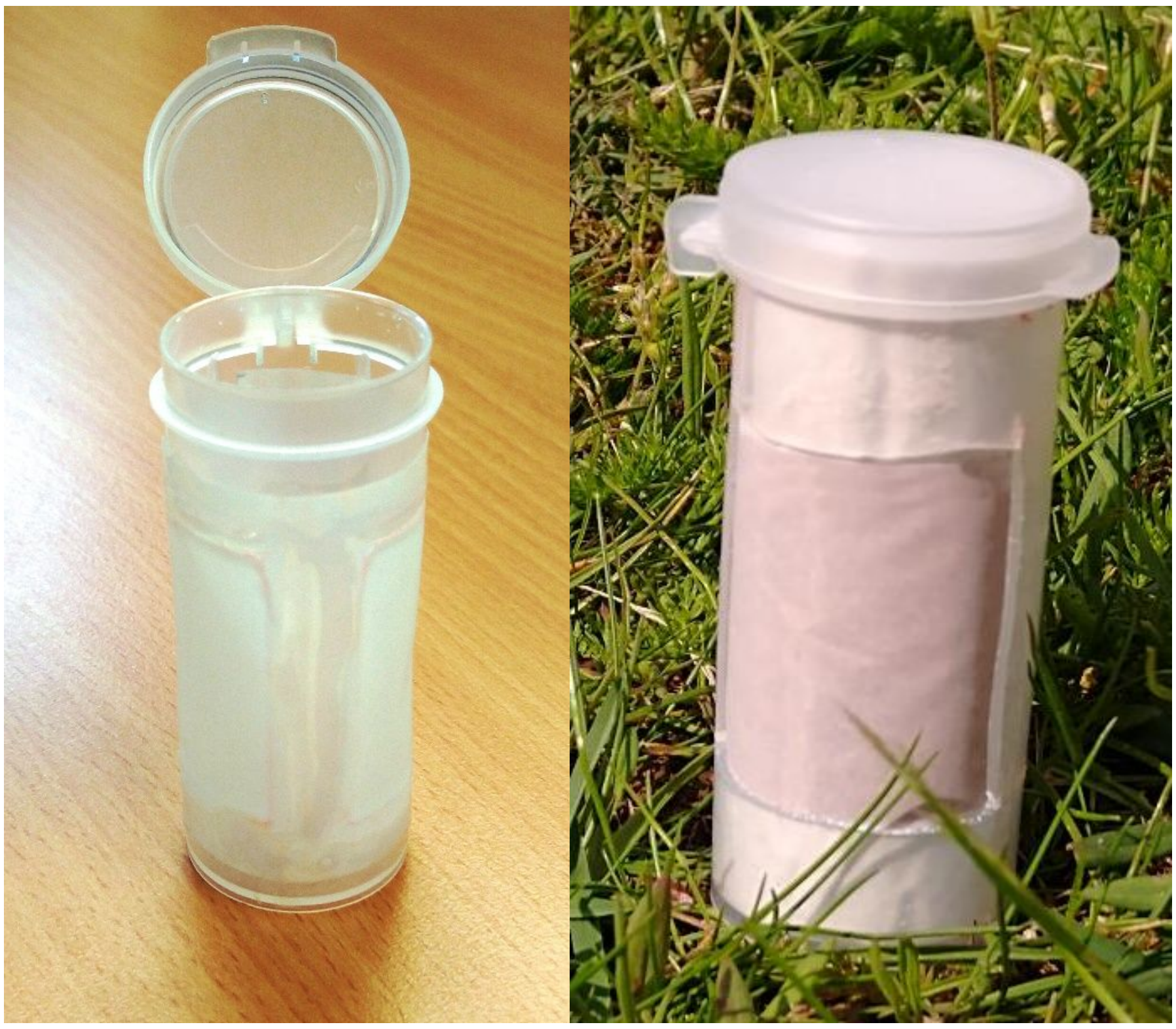

Figure S4. Left: Empty membrane cylinders. Right: Filled membrane cylinders

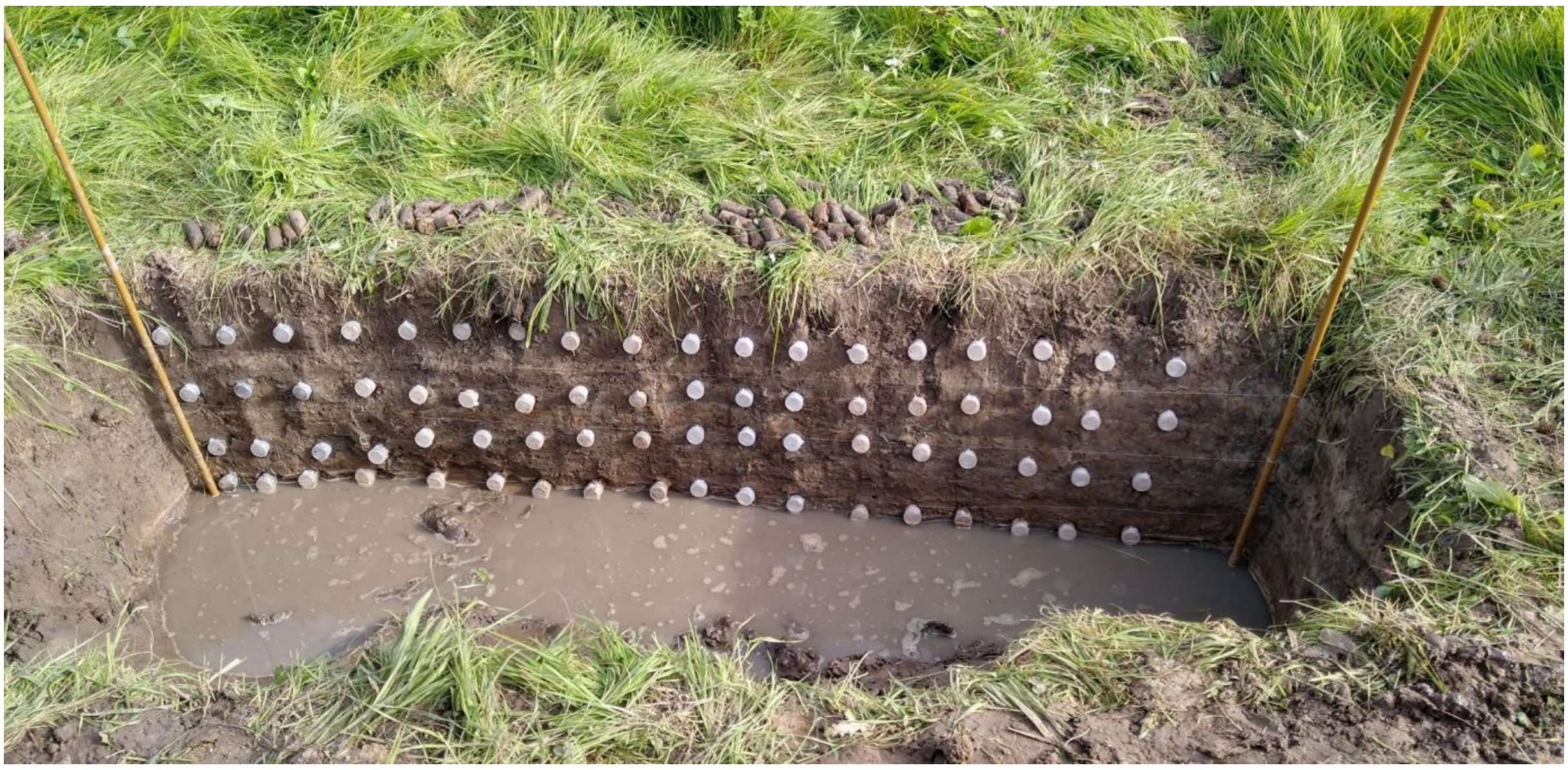

Figure S5a. Krautsand high marsh profile at the beginning of the field incubation. Filled membrane cylinders are inserted horizontally at 15, 30, 45 and 60 cm depths.

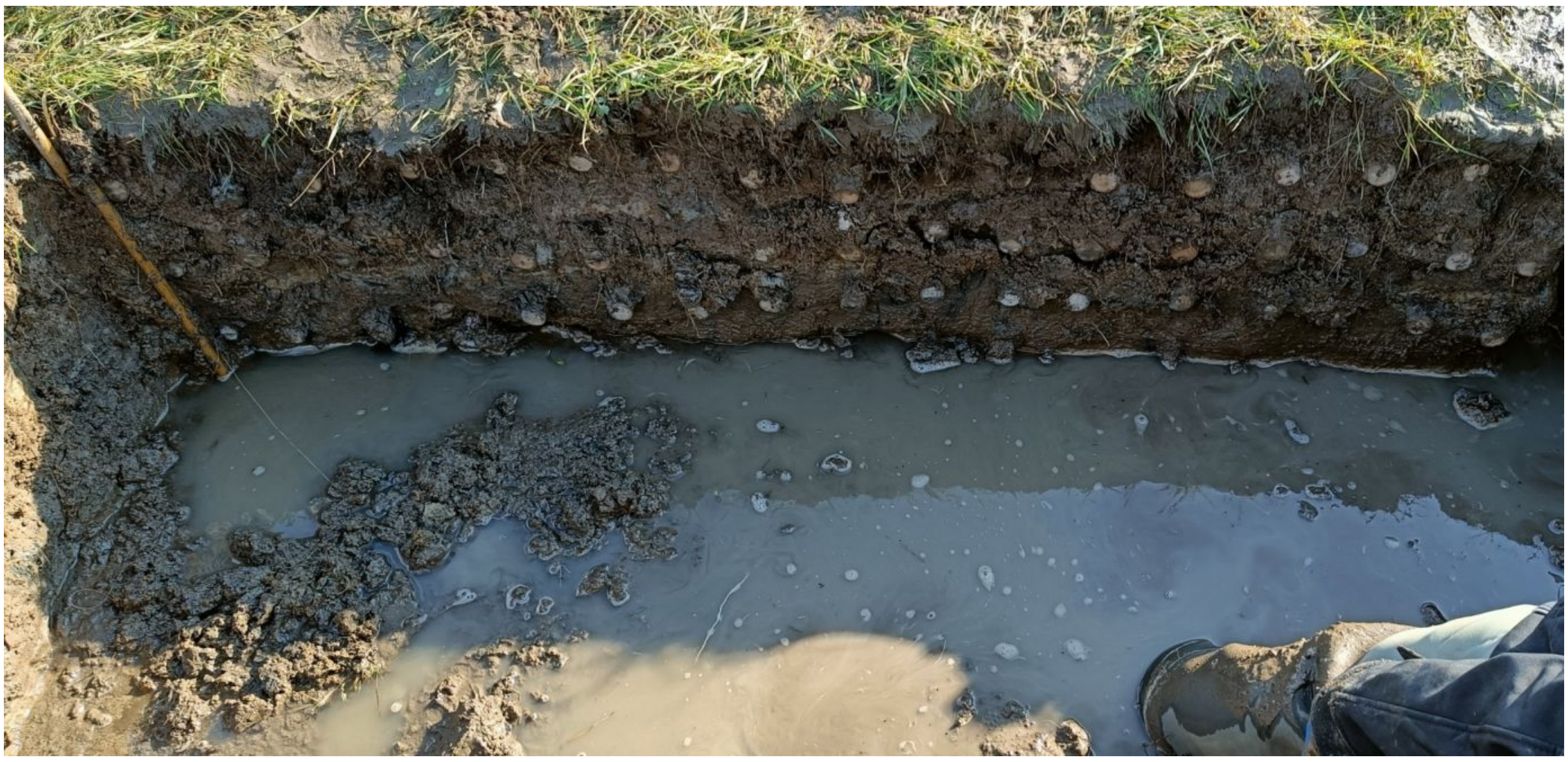

Figure S5b. Krautsand high marsh profile at the end of the field incubation.

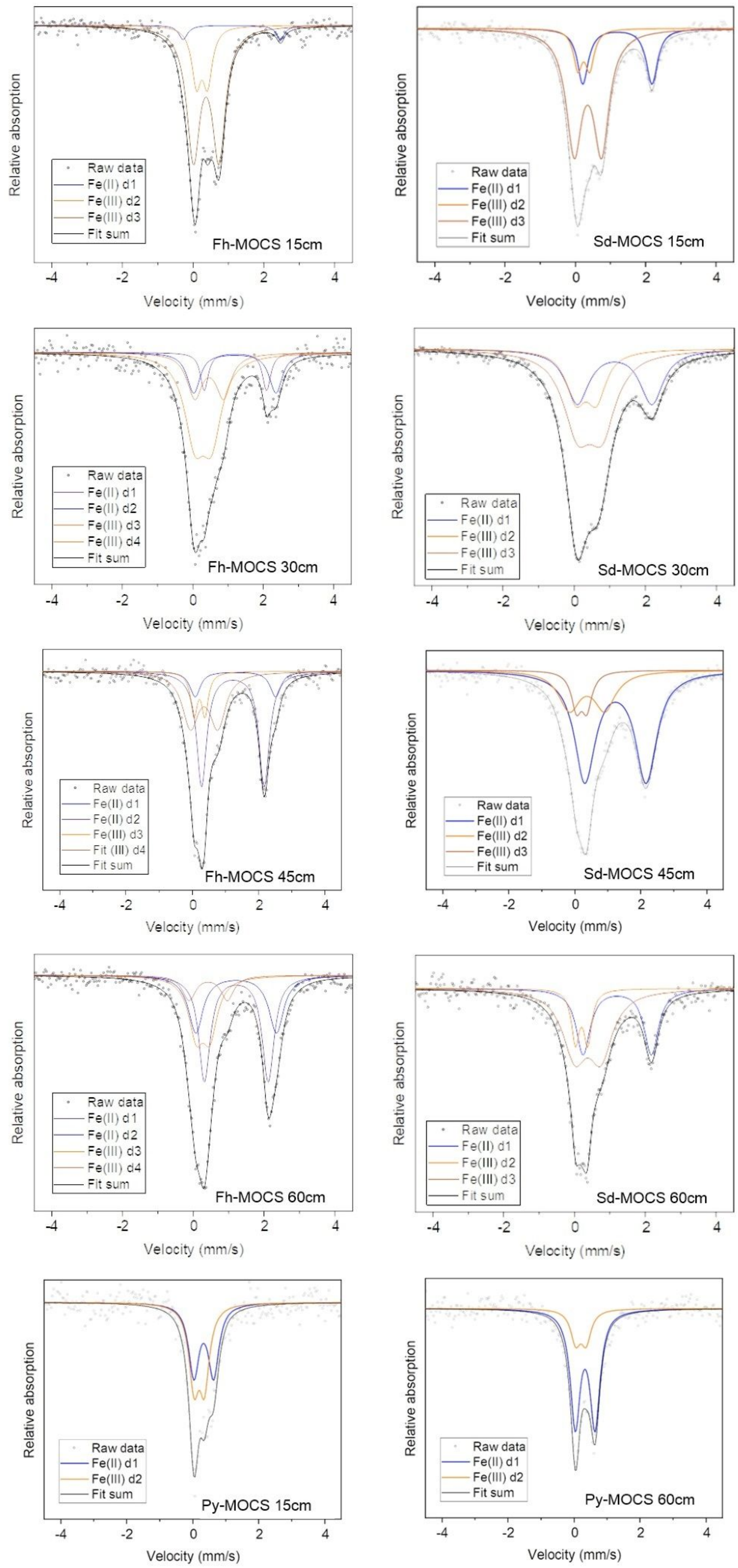
Relative absorption
Velocity (mm/s)
Raw data
Fe(II) d1
Fe(III) d2
Fe(III) d3
Fit sum
Fh-MOCS 15cm
Sd-MOCS 15cm
Fe(II) d2
Fe(III) d4
Fh-MOCS 30cm
Sd-MOCS 30cm
Fit (III) d4
Fh-MOCS 45cm
Sd-MOCS 45cm
Fh-MOCS 60cm
Sd-MOCS 60cm
Py-MOCS 15cm
Py-MOCS 60cm

Figure S6. Fitted room-temperature Mössbauer spectra of recovered Fe mineral–organic–clay–sand aggregates after six months of in situ incubation in tidal marsh soil. Spectra are shown for ferrihydrite-based aggregates (Fh-MOCS), siderite-based aggregates (Sd-MOCS) and for pyrite-based aggregates (Py-MOCS). Grey symbols indicate raw absorption data, colored lines represent fitted Fe sites, and the black line shows the summed fit. Fitted doublets are assigned to Fe(II) and Fe(III) environments based on their isomer shift and quadrupole splitting parameters.

Table S1. Isotopic composition and organic carbon content of pre-incubation materials used in the field experiment. Fe-MOCS refers to artificial Fe mineral-organic matter-clay-sand aggregates ferrihydrite (Fh), siderite (Sd) and and pyrite (Py). POM indicates added particulate organic matter (ground reed).

**Pre-incubation materials**	**$\delta^{13}C$ calibrated (‰)**	**C (%)**
reed unlabeled	-26.69	45.24
^{13}C-tracer labeled reed	258.39	41.55
^{13}C-tracer dissolved organic matter (dried)	471.83	n.a.
^{13}C-tracer labeled Fh-MOCS	321.34	0.10
^{13}C-tracer labeled Fh-MOCS + reed POM	-6.21	1.20
^{13}C-tracer labeled Py-MOCS	346.15	0.11
^{13}C-tracer labeled Py-MOCS + reed POM	7.59	1.32
^{13}C-tracer labeled Sd-MOCS + reed POM	-1.04	1.25

Table S2. Mössbauer spectroscopy parameters (Isomer shift δ and quadrupole splitting ΔEQ, in mm s^{-1}) of Fe mineral-organic-clay-sand of ferrihydrite, siderite and pyrite (Fh-MOCS, Sd-MOCS, Py-MOCS) incubated with organic carbon at different soil depths compared to literature (Murad 1988; Murad und Cashion 2004; Gil et al. 1992). Uncertainty is given in parentheses. *Manually fitted.

Sample		Phase 1		Phase 2		Phase 3		Phase 4	
	cm	δ	ΔE_Q	δ	ΔE_Q	δ	ΔE_Q	δ	ΔE_Q
Fh-	15	0.366(17)	0.722(42)			1.093(68)	2.75(13)	0.250(33)	0.319(60)
MOCS	30	0.46(58)	0.8(11)	1.213(50)	1.76(14)	1.19(34)	2.33(60)	0.29(26)	0.48(47)
	45	0.354(44)	0.824(13)	1.227(17)	1.888(42)	1.30(11)	2.42(25)	0.216(41)	0.338(98)
	60	0.421(80)	1.08(19)	1.218(19)	1.800(96)	1.20(10)	2.3(19)	0.30(11)	0.40(21)
Sd-	15	0.352(15)	0.776(45)	1.195(18)	1.963(37)			0.240(39)	0.340(69)
MOCS	30	0.44(36)	0.69(81)	1.13(64)	2.10(13)			0.33(32)	0.57(44)
	45	0.376(50)	0.73(19)	1.208(37)	1.926(81)			0.199(34)	0.353(68)
	60	0.372(56)	1.07(16)	1.234(23)	1.853(48)			0.207(60)	0.320(70)
Interpretation		ferrihydrite		siderite		illite Fe(II)		illite Fe(III)	
Literature		0.35-0.38	0.51-0.9	1.20-1.26	1.75-1-82	0.95-1.47	2.38-2.88	0.26-0.38	0.18-0.88
Not clearly interpretable phases									
Py-	15	0.33(65)	0.6(12)					0.19(55)	0.3(12)
MOCS	60	0.334*	0.6*					0.207*	0.32*

Table S3. Mössbauer spectroscopy parameters: Isomer shift δ, quadrupole splitting ΔE_Q and site population of Fe mineral-organic-clay-sand of ferrihydrite, siderite and pyrite (Fh-MOCS, Sd-MOCS, Py-MOCS). Uncertainty is given in parentheses. *Manually fitted due to parameter overlap.

	**Signal**	**Δ (mm s^{-1})**	**ΔE_Q (mm s^{-1})**	**Site Population (%)**
Fh-MOCS-15cm	Fe(II) d1	1.093(68)	2.75(13)	5.4(31)
	Fe(III) d2	0.366(17)	0.722(42)	73(11)
	Fe(III) d3	0.250(33)	0.319(60)	21.9(97)
Fh-MOCS-30cm	Fe(II) d1	1.19(34)	2.33(60)	18(22)
	Fe(II) d2	1.213(50)	1.76(14)	10(18)
	Fe(III) d3	0.29(26)	0.48(47)	51(58)
	Fe(III) d4	0.46(58)	0.8(11)	21(55)
Fh-MOCS-45cm	Fe(II) d1	1.30(11)	2.42(25)	10(10)
	Fe(II) d2	1.227(17)	1.888(42)	46(10)
	Fe(III) d3	0.216(41)	0.338(98)	10.5(48)
	Fe(III) d4	0.354(44)	0.824(13)	33.2(66)
Fh-MOCS-60cm	Fe(II) d1	1.2(10)	2.3(19)	26(31)
	Fe(II) d2	1.218(19)	1.800(96)	39(29)
	Fe(III) d3	0.3(11)	0.4(21)	25(13)
	Fe(III) d4	0.421(80)	1.08(19)	10(12)
Sd-MOCS-15cm	Fe(II) d1	1.195(18)	1.963(37)	22.0(31)
	Fe(III) d2	0.240(39)	0.340(69)	12.0(69)
	Fe(III) d3	0.352(15)	0.776(45)	66.1(86)
Sd-MOCS-30cm	Fe(II) d1	1.13(64)	2.1(13)	29.8(34)
	Fe(III) d2	0.33(32)	0.57(44)	22(88)
	Fe(III) d3	0.44(36)	0.69(81)	49(87)
Sd-MOCS-45cm	Fe(II) d1	1.208(37)	1.926(81)	32.7(53)
	Fe(III) d2	0.199(34)	0.353(68)	14.5(95)
	Fe(III) d3	0.376(50)	0.73(19)	53(14)
Sd-MOCS-60cm	Fe(II) d1	1.234(23)	1.853(48)	66.2(49)
	Fe(III) d2	0.372(56)	1.07(16)	23.3(87)
	Fe(III) d3	0.207(60)	0.320(70)	10.5(53)
Py-MOCS-15cm	Fe(II) d1	0.33(65)	0.6(12)	49(52)
	Fe(III) d2	0.19(55)	0.3(12)	51(53)
Py-MOCS-60cm	Fe(II) d1	0.334052*	0.6*	78.3(57)
	Fe(III) d2	0.206838*	0.32*	21.7(51)

Table S4. Test of Fe extraction efficiency by sequential extraction according to Lenstra et al. (2019; 2021). MLR = Mineralmühle Leun, Rau Gmbh (Leun, Germany), LAN = LANXESS Deutschland GmbH (Köln, Germany), syn. = synthesized

Fe mineral	Extraction efficiency					Supplier or synthesis method
	Fe_{Asc}	Fe_{HCl}	Fe_{Dith}	Fe_{Oxa}	Fe_{HNO3}	
$FeSO_4 \cdot 7H_2O$	Target					VWR
Ferrihydrite	Target					Schwertmann and Cornell (2000)
Schwertmannite	Target					Regenspurg et al. (2004)
Vivianite	Target					Eynard et al. (1992)
Mackinawite	Moderate	Low				Lennie et al. (1995)
Jarosite	Low	Moderate				Baron and Palmer (1996)
Siderite natural		Target	Marginal			MLR Eisenspat 52
Siderite syn.		Target				Poulton and Canfield (2005)
Lepidocrocite	Marginal	Marginal	(dark grey)			Schwertmann and Cornell (2000)
Goethite brown			Target	Marginal		LAN Bayoxide E 216
Goethite yellow			Target	Marginal		LAN Bayferrox 920Z
Hematite			Target			MLR Eisenglanz 56
Magnetite			Marginal	Moderate		MLR Magnetit 48
Pyrite					Target	MLR Schwefeleisen
Target	100-85%		(black)			
Moderate	85-50		(dark grey)			
Low	50-25%		(grey)			
Marginal	25-5%		(light grey)			
Limited	<5%		(white)			

Table S5. Concentrations of dissolved constituents in soil porewater (60 cm depth) extracted by centrifugation.

Ion species	concentration
	mmol l^{-1}
**Cl^-**	11.867
**Br^-**	0.011
**NO_3^{2-}**	0.276
**$H_2PO_4^-/HPO_4^{2-}$**	0.002
**SO_4^{2-}**	1.378
**Na^+**	12.897
**K^+**	0.033
**Mg^{2+}**	0.367
**Ca^{2+}**	2.254
**HCO_3^-**	4.710
